# MMP-9 regulates disulphide isomerase activity of TGM2 to enhance fusion glycoprotein-mediated syncytium formation of respiratory syncytial virus

**DOI:** 10.1093/procel/pwaf063

**Published:** 2025-08-11

**Authors:** Bao Xue, Anqi Zhou, Yihang Zhong, Yuhan Mao, Ran Peng, Yuhang Chen, Jiayi Zhong, Junjun Liu, Yuan Zhou, Yuying Fang, Wei Zhang, Jielin Tang, Wei Peng, Jia Liu, Qi Yang, Xinwen Chen

**Affiliations:** Key Laboratory of Virology and Biosafety, Wuhan Institute of Virology, Chinese Academy of Sciences, Wuhan 430071, China; Guangzhou National Laboratory, Guangzhou 510005, China; College of Life Sciences, University of Chinese Academy of Sciences, Beijing 100049, China; Guangzhou National Laboratory, Guangzhou 510005, China; State Key Laboratory of Respiratory Disease, Guangzhou Medical University, Guangzhou 511436, China; Guangzhou National Laboratory, Guangzhou 510005, China; Guangzhou National Laboratory, Guangzhou 510005, China; Guangzhou National Laboratory, Guangzhou 510005, China; State Key Laboratory of Respiratory Disease, Guangzhou Medical University, Guangzhou 511436, China; Department of Infectious Diseases, Union Hospital, Tongji Medical College, Huazhong University of Science and Technology, Wuhan 430022, China; Guangzhou National Laboratory, Guangzhou 510005, China; Guangzhou National Laboratory, Guangzhou 510005, China; Key Laboratory of Virology and Biosafety, Wuhan Institute of Virology, Chinese Academy of Sciences, Wuhan 430071, China; Guangzhou National Laboratory, Guangzhou 510005, China; Guangzhou National Laboratory, Guangzhou 510005, China; Guangzhou National Laboratory, Guangzhou 510005, China; Guangzhou National Laboratory, Guangzhou 510005, China; Guangzhou National Laboratory, Guangzhou 510005, China; Department of Infectious Diseases, Union Hospital, Tongji Medical College, Huazhong University of Science and Technology, Wuhan 430022, China; Guangzhou National Laboratory, Guangzhou 510005, China; State Key Laboratory of Respiratory Disease, Guangzhou Medical University, Guangzhou 511436, China; Key Laboratory of Virology and Biosafety, Wuhan Institute of Virology, Chinese Academy of Sciences, Wuhan 430071, China; Guangzhou National Laboratory, Guangzhou 510005, China; State Key Laboratory of Respiratory Disease, Guangzhou Medical University, Guangzhou 511436, China

**Keywords:** respiratory syncytial virus, fusion glycoprotein, matrix metalloproteinase 9, transglutaminase 2, protein disulfide isomerase

## Abstract

Respiratory syncytial virus (RSV) exploits host proteases to enhance its replication efficiency; however, the precise mechanisms remain unclear. Through high-throughput screening, we identified four matrix metalloproteinase 9 (MMP-9) inhibitors (including JNJ0966 and doxycycline hyclate) that suppress RSV infection *in vitro* and *in vivo*. Mechanistic studies revealed a proteolytic cascade wherein MMP-9 cleaves transglutaminase 2 (TGM2) at the PVP^375^↓VR site, generating an N-terminal fragment (1–375) that activates its protein disulfide isomerase (PDI) activity. This TGM2-dependent PDI activity catalyzes disulfide bond rearrangement in the RSV fusion glycoprotein (F), enabling F protein maturation, a prerequisite for membrane fusion and syncytium formation—key processes driving late-stage viral propagation. Genetic ablation of MMP-9 significantly attenuated RSV infectivity, while pharmacological inhibition reduced pulmonary viral loads and mitigated lung pathology in infected mice. Our study defines a unified MMP-9→TGM2→F axis as the core mechanism driving RSV replication and validates MMP-9 as a therapeutic target.

## Introduction

Respiratory syncytial virus (RSV) is a leading global cause of severe respiratory disease in infants, elderly patients, and immunocompromised patients (Ruiz-Galiana et al., 2024). Despite its substantial health burden, therapeutic options remain limited. Currently approved treatments include the monoclonal antibody palivizumab and the antiviral ribavirin; however, both are associated with significant limitations (Fenton et al., 2004; Gavin and Katz, 2002; Hammitt et al., 2022; Helmink et al., 2016). Recent advancements, including the approval of nirsevimab and two vaccines, have expanded prevention strategies (Hammitt et al., 2022; Kampmann et al., 2023; Papi et al., 2023; Walsh et al., 2023). Nevertheless, there remains an urgent need for effective small-molecule drugs to address symptomatic RSV infections.

RSV is a non-segmented, negative-sense, single-stranded RNA virus belonging to the family *Pneumoviridae* (genus *Orthopneumovirus*) (Collins and Graham, 2008; Kaler et al., 2023; Duan et al., 2024; Yang et al., 2024). Its genome is composed of approximately 15,222 nucleotides and 10 genes that encode 11 proteins, including the fusion (F) protein, which is a critical determinant of viral pathogenicity (Royen et al., 2022). The F protein facilitates both virus–cell and cell–cell membrane fusion, processes that are essential for viral entry and syncytium formation (Mammas et al., 2020). Host furin-like proteases cleave the F protein at two sites (R109 and R136), releasing a 27-amino acid peptide (p27) and generating F1 and F2 subunits linked by disulfide bonds (C37–C439, C69–C212) (Day et al., 2006; Ye et al., 2020). These subunits subsequently assemble into a mature trimeric F protein, which is trafficked to the cell surface and drives syncytium formation, a process critical for viral spread and pathogenesis, which is believed to be important for both pathogenicity and transmission of the virus (Shaikh et al., 2012).

Host proteases play pivotal roles in viral infections by modulating viral entry, replication, and immune evasion (Li et al., 2012). For example, TMPRSS2 facilitates the cleavage of the SARS-CoV-2 spike protein, thereby enabling viral entry (Wulandari et al., 2021), and serves as the functional receptor for human coronavirus HKU1 (Saunders et al., 2023). Caspase-6 cleaves coronavirus nucleocapsid proteins to antagonize interferon responses (Chu et al., 2022). In addition, matrix metalloproteinases (MMPs), calcium-dependent zinc-endopeptidases of the metzincin superfamily (Devereux et al., 2014; Kong et al., 2009; Ma et al., 2014), are implicated in viral infections (Bhardwaj and Singh, 2023; Guo et al., 2022; Talmi-Frank et al., 2016). As previously reported, membrane type 1 matrix metalloproteinase (MT1-MMP) promotes SARS-CoV-2 cell entry by releasing soluble ACE2 (Guo et al., 2022). MMP-9 enhances hepatitis B virus replication by suppressing interferon signaling (Chen et al., 2017). ZIKV infection upregulates MMP-9, stabilized by NS1-induced K63-linked polyubiquitination, disrupting BTB and promoting testicular entry (Hui et al., 2020). MMP-9 has been associated with RSV replication, but the underlying mechanisms remain poorly defined (Kong et al., 2015; Xu et al., 2021).

TGM2, a member of the transglutaminase (TGase) family of enzymes (EC 2.3.2.13), catalyzes Ca^2+^-dependent protein crosslinking and exhibits GTP/ATPase and protein disulfide isomerase (PDI) activities (Hasegawa et al., 2003; Lorand and Graham, 2003; Nakano et al., 2010; Takeuchi et al., 1994). TGM2 is involved in many biological processes, including inflammation, wound healing, and apoptosis (Mehta and Han, 2011; Telci and Griffin, 2006; Zhang et al., 2016), and has been implicated in diseases such as celiac disease, neurodegenerative disorders, and cancer (Ashour et al., 2014; Klöck et al., 2012; Oono et al., 2014). Recent studies have demonstrated that SARS-CoV-2 and Dengue virus infections upregulate TGM2, implicating its potential role in viral infection (Jiang and Sun, 2020; Shaath et al., 2020); however, its involvement in RSV pathogenesis remains unexplored.

We screened a library of 246 protease inhibitors and identified four MMP-9-targeting compounds that effectively inhibit RSV infection. MMP-9 cleaves TGM2 into two fragments, thereby enhancing the PDI activity of the N-terminal fragment, which promotes the maturation of the RSV F protein and syncytium formation. Treatment with the MMP-9 inhibitor JNJ0966 significantly reduced RSV replication in both HBEC and RSV-infected mice. Moreover, MMP-9-deficient mice exhibited reduced susceptibility to RSV infection. These findings reveal MMP-9’s critical role in RSV infection and its potential as a therapeutic target.

## Results

### Discovery and identification of metalloproteinase inhibitors against RSV infection

**Figure 1.**
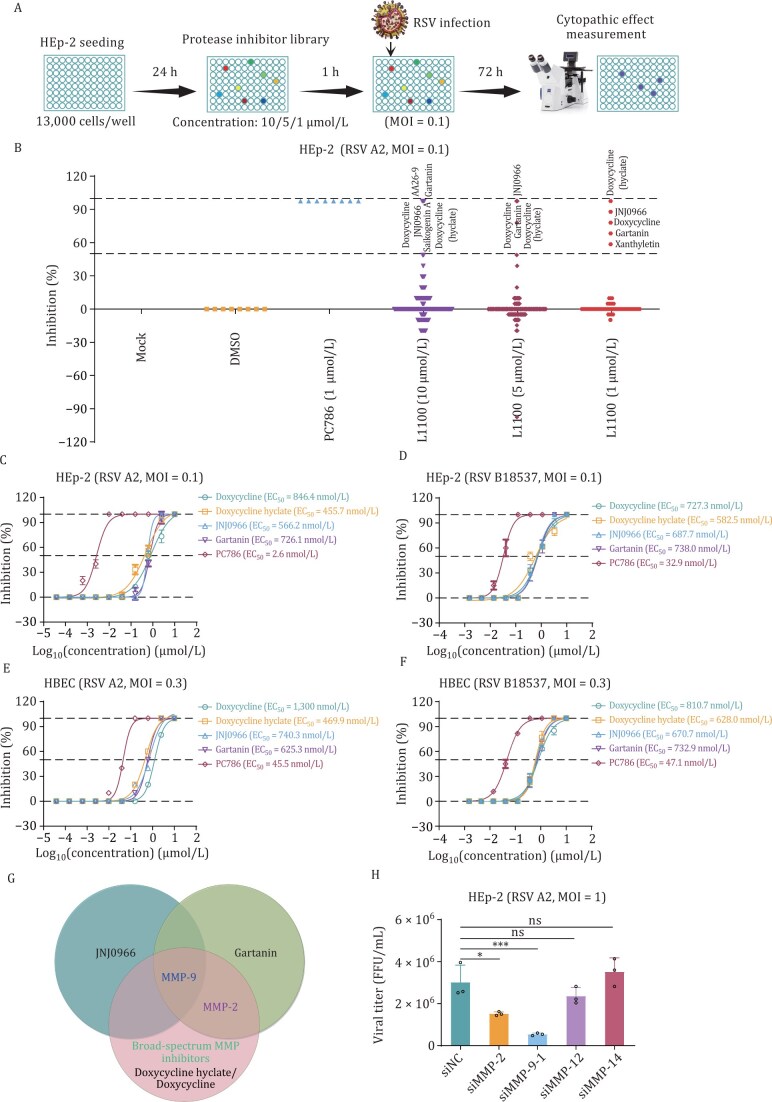
Antiviral activity of metalloproteinase inhibitors against RSV ***in vitro***. (A) Experimental workflow for RSV A2 infection and compound treatment. (B) High-throughput screening of TargetMol L1100 library (246 host protease inhibitors targeting 10 pathways: MMPs, caspases, proteasomes, apoptosis regulators, cysteine proteases, gamma-secretase, autophagy modulators, serine proteases, DPP-4, and beta-secretase) using concentration gradients (1, 5, 10 µmol/L). HEp-2 cells were pretreated with compounds for 1 hour prior to RSV A2 infection (MOI = 0.1). Plaque formation was quantified at 72 hours post-infection (hpi) and normalized to DMSO controls. (C–F) Dose-dependent inhibition curves for metalloproteinase inhibitors (doxycycline, doxycycline hyclate, JNJ0966, gartanin) and positive control PC786 (RSV L-protein inhibitor) against RSV genotypes in HEp-2 and HBEC models. mean ± SD, *n* = 3. (G) Target overlap analysis of four MMP-9-targeting inhibitors. (H) Viral titers in supernatants of HEp-2 cells transfected with MMP-specific siRNAs (24 h) followed by RSV A2 infection (MOI = 1, 72 h). mean ± SD, *n* = 3. The results are representatively shown with three random experiments. The error bars indicate the mean ± SD of three technical replicates (*n* = 3). Statistical significance was determined by one-way ANOVA with Dunnett’s post hoc test: *P < 0.05, ***P < 0.001; ns, not significant.

To identify host protease involved in RSV infection, we conducted a high-throughput screening of a library of 246 protease inhibitors targeting 10 signaling pathways, including MMP, caspase, and cysteine protease, etc. (Fig. 1A and 1B). The results showed that four compounds—doxycycline, doxycycline hyclate, JNJ0966, and gartanin—exhibited significant dose-dependent inhibition of RSV infection in HEp-2 cells (Figs. 1B and S1A). These compounds demonstrated potent antiviral activity against both RSV A2 and B18537 strains, with EC_50_ values ranging from 455.7 to 846.4 nmol/L and minimal cytotoxicity (CC_50_ > 20 µmol/L) (Figs. 1C, 1D, and S1B). Similar antiviral efficacy was observed in primary human bronchial epithelial cells (HBEC), with EC_50_ values ranging from 469.9 to 1,300 nmol/L (Figs. 1E, 1F, and S1C).

Cluster analysis revealed that all four compounds target MMP-9 (Luo et al., 2017; Patnaik et al., 2021; Samtani et al., 2009; Scannevin et al., 2017) (Fig. 1G). To further validate the critical role of MMP-9, we conducted knockdown experiments for MMP-2, MMP-9, MMP-12, and MMP-14 in HEp-2 cells using siRNAs. The results showed that both MMP-9 and MMP-2 knockdowns reduced RSV replication; however, the knockdown of MMP-9 exhibited significantly stronger inhibition (*P* < 0.001) compared to MMP-2 (Figs. 1H, S1D, and S1E). These findings collectively suggest that MMP-9 plays a pivotal role as a host factor in RSV infection.

### MMP-9 is required for RSV infection at a late stage

**Figure 2.**
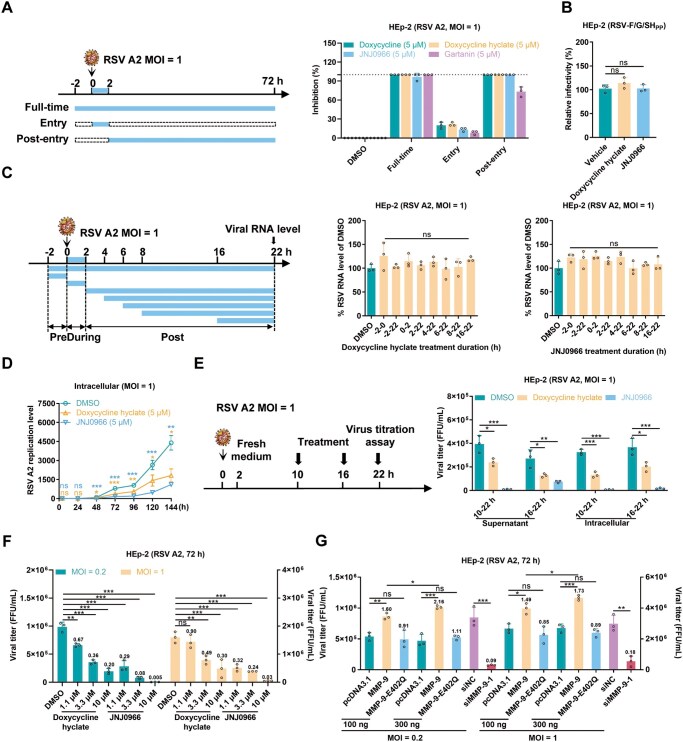
MMP-9 regulates late-stage RSV infection. (A) Time-of-addition assay design (left). HEp-2 cells were treated with metalloproteinase inhibitors under three regimens: “Full-time” (2 h pretreatment + continuous exposure), “Entry” (viral attachment phase only), or “Post-entry” (≥ 2 hpi). Cells infected with RSV A2 (MOI = 1) were analyzed for cytopathic effect inhibition at 72 hpi versus DMSO controls. mean ± SD, *n* = 3. (B) No inhibition of RSV F/G/SH pseudovirus entry by doxycycline hyclate or JNJ0966 (5 µmol/L). mean ± SD, *n* = 3. (C) Temporal sensitivity analysis (left). Viral RNA levels (22 hpi) in HEp-2 cells treated with 5 µmol/L inhibitors at defined intervals: pre-infection (−2–0 h), entry (0–2 h), or post-entry (2–16 h) phases (right). mean ± SD, *n* = 3. (D) Multicycle replication kinetics. Intracellular RSV RNA (fusion gene copies) in HEp-2 cells treated with 5 µmol/L inhibitors at indicated time points (4–144 hpi). mean ± SD, *n* = 3. (E) Viral assembly/release inhibition assay. Supernatant viral titers at 22 hpi after post-entry treatment (10/16 hpi) (right). mean ± SD, *n* = 3. (F) Dose-dependent suppression of RSV progeny (MOI = 0.2/1) by inhibitors (72 hpi), quantified by FFA. mean ± SD, *n* = 3. (G) Viral titers in HEp-2 cells overexpressing wild-type MMP-9, catalytically inactive mutant (E402Q), or MMP-9-knockdown (siMMP-9) models (72 hpi, MOI = 0.2/1). mean ± SD, *n* = 3. The results are representatively shown with three random experiments. The error bars indicate the mean ± SD of three technical replicates (*n* = 3). Statistical analysis: one-way ANOVA with Dunnett’s post hoc test (B–G); unpaired two-tailed Student’s *t*-test (G). *P < 0.05, **P < 0.01, ***P < 0.001; ns, not significant. Column labels indicate fold-changes versus controls.

To ascertain the timing of compound action in inhibiting RSV infection, we performed time-of-addition assays (Fig. 2A). Adding the compounds during the first 2 hours of infection did not inhibit RSV. Still, administration 2 hours post-infection (hpi) or throughout the infection period showed equivalent inhibition, indicating post-entry activity. Subsequently, we selected doxycycline hyclate, a broad-spectrum MMP inhibitor (Patnaik et al., 2021; Samtani et al., 2009), and JNJ0966, which specifically targets MMP-9 (Scannevin et al., 2017), for further experimental investigation. The above result was confirmed by an RSV pseudovirus assay, which demonstrated no impact on viral entry (Fig. 2B). RT-qPCR analysis further revealed that the compounds did not affect intracellular viral RNA levels, regardless of the timing or duration of administration (Fig. 2C), suggesting that they do not interfere with viral RNA synthesis but instead act at a later stage in the RSV life cycle. Multistep growth curve analyses revealed that the compounds reduced viral RNA levels starting at 48 hpi (Fig. 2D), suggesting that they modulate viral spread rather than the early stages of replication.

To evaluate the effects of the compounds on RSV assembly and release, we treated HEp-2 cells with the inhibitors at 10 hpi (the onset of virion assembly) or 16 hpi (the onset of virion release) following RSV infection (Malhi et al., 2021). Supernatants were collected at 22 hpi for viral titer quantification. The results demonstrated that the compounds significantly inhibited viral assembly or release (Fig. 2E). Furthermore, at 72 hpi, viral titers were quantified following infection with varying doses of RSV (0.2 and 1 MOI). Compound doxycycline hyclate and JNJ0966 exhibited dose-dependent inhibition of RSV replication (Fig. 2F). Consistent with these findings, siRNA-mediated knockdown of MMP-9 resulted in a substantial reduction in viral titers (Fig. 2G). Subsequently, overexpression of wild-type (WT) MMP-9 significantly enhanced RSV replication, whereas the enzymatically inactive mutant (E402Q) (Roderfeld et al., 2006; Rotenberg et al., 2024; Rowsell et al., 2002; Tochowicz et al., 2007) failed to produce a similar effect (Fig. 2G). These results collectively demonstrate that MMP-9’s protease activity is essential for promoting RSV spread.

### MMP-9 promotes RSV F protein-mediated syncytia formation

**Figure 3.**
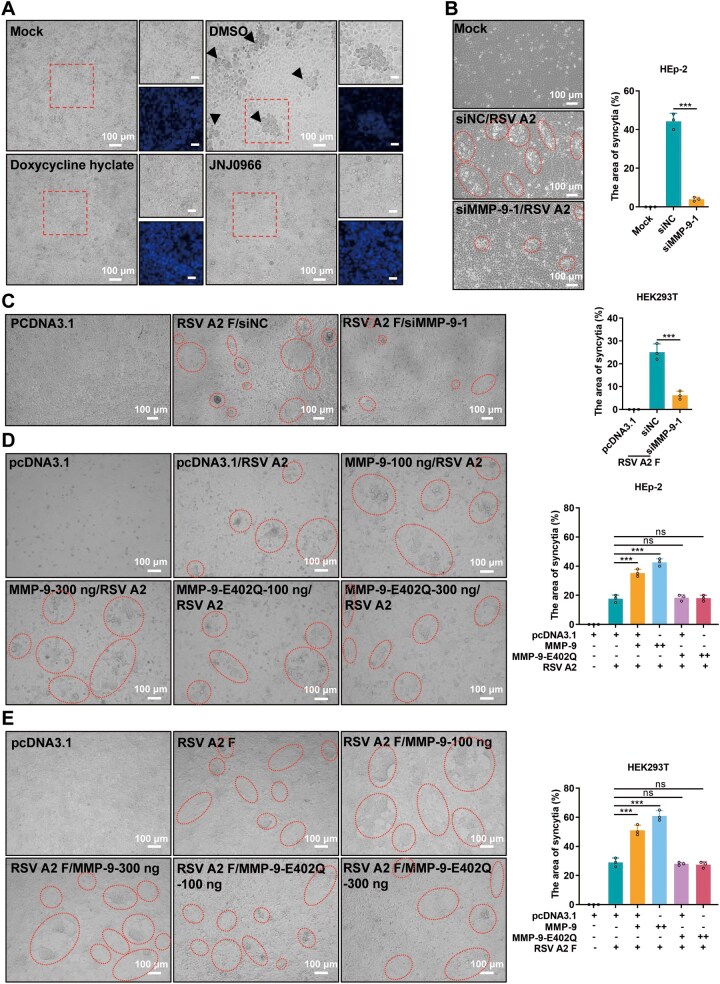
MMP-9 facilitates RSV F protein-mediated cell-cell fusion. (A) Syncytium formation in HEp-2 cells pretreated with 5 µmol/L doxycycline hyclate or JNJ0966 for 1 hour prior to RSV A2 infection (MOI = 0.2, 72 hpi). Representative phase-contrast images (10×) show syncytia (black arrows). Boxed regions depict DAPI-stained nuclei. Scale bar: 100 µm. (B) MMP-9 knockdown suppresses syncytium formation. HEp-2 cells transfected with siMMP-9 (24 h) were infected with RSV A2 (MOI = 0.2, 72 hpi). Syncytia (circles) were quantified by ImageJ software. mean ± SD, *n* = 3. (C) MMP-9 knockdown suppresses syncytium formation in HEK293T cells co-transfected with siNC/siMMP-9 (20 nmol/L) and RSV A2 F plasmid (500 ng). Scale bar: 100 µm. Syncytia (circles) were quantified 48 hours post-transfection. mean ± SD, *n* = 3. (D) Enhanced syncytogenesis by MMP-9 overexpression. HEp-2 cells transfected with wild-type MMP-9 or catalytically inactive mutant (E402Q; 100/300 ng) were infected with RSV A2 (MOI = 0.2, 72 hpi). Syncytia (circles) were quantified. mean ± SD, *n* = 3. (E) MMP-9-dependent syncytium formation in HEK293T cells co-transfected with MMP-9/E402Q (100/300 ng) and RSV A2 F plasmid (500 ng). Scale bar: 100 µm. Syncytia (circles) were quantified 48 hours post-transfection. mean ± SD, *n* = 3. Images represent three biological replicates comprising nine fields each. The error bars indicate the mean ± SD of three biological replicates. Statistical significance was determined by unpaired two-tailed Student’s *t*-test (B and C); one-way ANOVA with Dunnett’s post-test (D and E). ***P < 0.001; ns, not significant.

RSV infection induces syncytium formation, a critical process for viral spread, resulting in significant cytopathic effect (CPE) characterized by the formation of large, multi-nucleated syncytia in HEp-2 cells. Treatment with MMP-9 inhibitors or siRNA-mediated knockdown of MMP-9 significantly reduced syncytium formation (Figs. 3A–C, S2A and S2B), whereas overexpression of MMP-9 enhanced this process (Figs. 3D, 3E, S2C, and S2D). Importantly, WT MMP-9, but not the enzymatically inactive mutant E402Q, promoted syncytium formation induced by RSV or RSV F proteins from different subtypes (Figs. 3D, 3E, S2C, and S2D). These findings confirm that MMP-9’s enzymatic activity is essential for facilitating RSV-induced syncytium formation.

**Figure 4.**
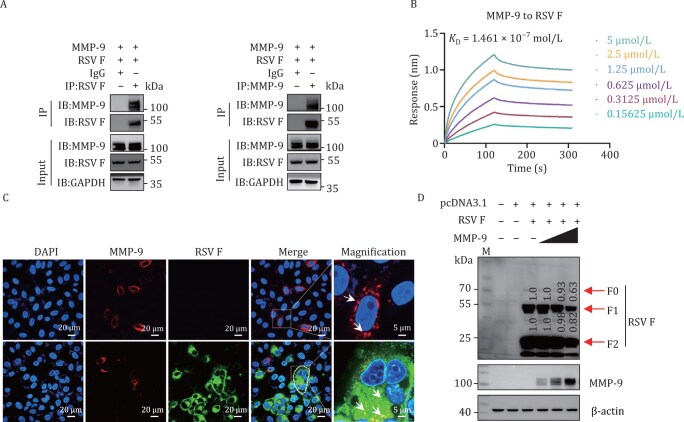
MMP-9 interacts with RSV F protein. (A) MMP-9–F protein interaction analysis. HEK293T cells co-transfected with MMP-9 and RSV F plasmids (48 h) were subjected to co-immunoprecipitation (Co-IP) using anti-F, anti-MMP-9, or IgG control antibodies, followed by immunoblotting with indicated probes. Blots represent three independent experiments. (B) Binding affinity quantification. Biolayer interferometry (BLI) assays using ForteBio Octet measured DS-Cav1 (RSV F prefusion conformation) and MMP-9 interactions. The results represent three biological replicates. (C) Subcellular co-localization. Confocal microscopy of HEp-2 cells transfected with MMP-9 (24 h) ± RSV A2 infection (MOI = 1, 72 hpi). Nuclei (DAPI), MMP-9, and RSV F signals are shown. Scale bar: 20 µm. Images represent three biological replicates. (D) No effect on F protein proteolytic processing. Western blot of RSV F cleavage status in HEK293T cells co-expressing MMP-9 (100–500 ng) and RSV F (500 ng) for 48 h. β-Actin served as loading control. Blots represent three independent experiments.

Given the established role of MMP-9 in regulating RSV F protein-induced syncytium formation, we systematically investigated the molecular interaction between MMP-9 and the RSV F protein. Co-immunoprecipitation (Co-IP) and biolayer interferometry (BLI) assays revealed a strong interaction between MMP-9 and RSV F protein, with a dissociation constant (*K*_D_) of 1.461 × 10^−7^ mol/L (Figs. 4A, 4B, S3A, and S3B). Confocal microscopy further confirmed partial co-localization of MMP-9 and RSV F protein in RSV-infected cells (Fig. 4C). However, MMP-9 did not cleave RSV F protein, as demonstrated by immunoblotting assays (Fig. 4D), indicating that its role in syncytium formation is mediated through an indirect mechanism rather than direct proteolytic processing of the F protein.

### TGM2 interacts with MMP-9 to regulate RSV infection

**Figure 5.**
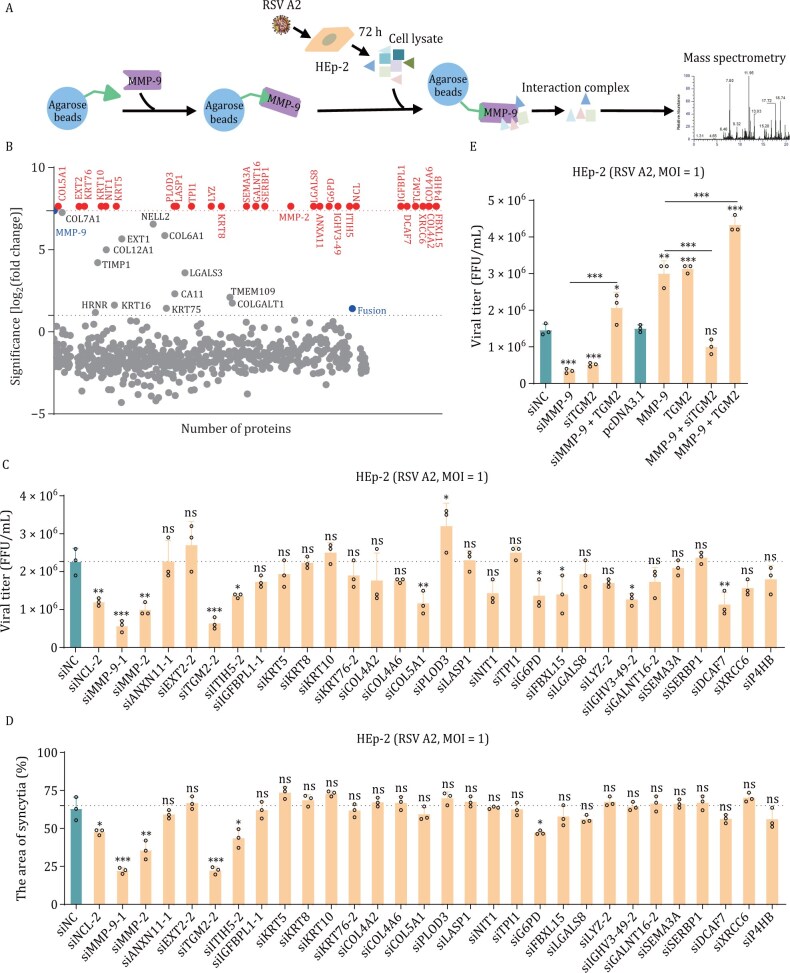
MMP-9 cooperates with TGM2 to modulate RSV infection. (A) MMP-9 interactome profiling strategy. Schematic of His-tag pull-down assay combined with RSV A2-infected HEp-2 lysates. (B) Proteomic screening. Scatter plot showing log_2_(fold changes) (log_2_FC) of 763 proteins co-purified with MMP-9-His versus beads-only control (MS analysis). Points labeled: 42 candidates (log_2_FC > 1). Blue: MMP-9 and RSV F (internal controls). Red: 29 candidates (log_2_FC > 7.37, consistent with MMP-9) were selected for functional validation. (C) siRNA screen of candidate regulators. HEp-2 cells transfected with target-specific siRNAs (24 h) were infected with RSV A2 (MOI = 1, 72 hpi). Viral titers in supernatants were quantified by FFA. mean ± SD, *n* = 3. (D) Syncytogenesis suppression. siRNA-treated HEp-2 cells infected with RSV A2 (MOI = 1, 72 hpi). The syncytia area was quantified by ImageJ software. mean ± SD, *n* = 3. (E) MMP-9-TGM2 functional interplay. Viral titers (FFA) in HEp-2 cells co-transfected with siMMP-9, siTGM2, MMP-9/TGM2 overexpression plasmids, or combinatorial constructs (72 hpi, MOI = 1). mean ± SD, *n* = 3. The results are representatively shown with three random experiments. The error bars indicate the mean ± SD of three technical replicates (*n* = 3). Statistical analysis: one-way ANOVA with Dunnett’s post hoc test (C–E); unpaired two-tailed Student’s *t*-test (E). *P < 0.05, **P < 0.01, ***P < 0.001; ns, not significant.

To elucidate the mechanism by which MMP-9 promotes RSV syncytium formation, we identified host proteins interacting with MMP-9 following RSV infection in HEp-2 cells. Using a 6× His-tagged MMP-9 construct (MMP-9-6× His) combined with tandem affinity purification and mass spectrometry, we identified 42 host proteins interacting with MMP-9 (29 with Log_2_FC > 7.37 and 13 with Log_2_FC > 1 compared to controls), including TGM2, MMP-2, keratins, collagens, and other functional proteins (Fig. 5A and 5B). Meanwhile, we observed an interaction between MMP-9 and the F protein, aligning with previous findings (Fig. 5B).

We performed functional screening on 29 highly enriched proteins. The screening revealed that knockdown of MMP-9 and TGM2 (*P* < 0.001), NCL, MMP-2, COL5A1, and DCAF7 (*P* < 0.01), ITIH5, G6PD, FBXL15, and IGHV3-49 (*P* < 0.05) reduced RSV progeny titers (Figs. 5C and S4A). Similar to MMP-9, knockdown of TGM2 effectively inhibited RSV-induced syncytium formation (*P* < 0.001), while knockdown of MMP-2, ITIH5, and G6PD also reduced syncytium formation but to a lesser extent (Fig. 5D). It remains unclear whether TGM2 employs a mechanism similar to MMP-9 or collaborates with MMP-9 to regulate RSV replication. Further experiments confirmed a significant functional correlation between MMP-9 and TGM2, as evidenced by both knockdown and overexpression assays (Fig. 5E). These findings highlight TGM2 as a critical interactor of MMP-9 in regulating RSV infection and syncytium formation.

### The cleavage of TGM2 by MMP-9

**Figure 6.**
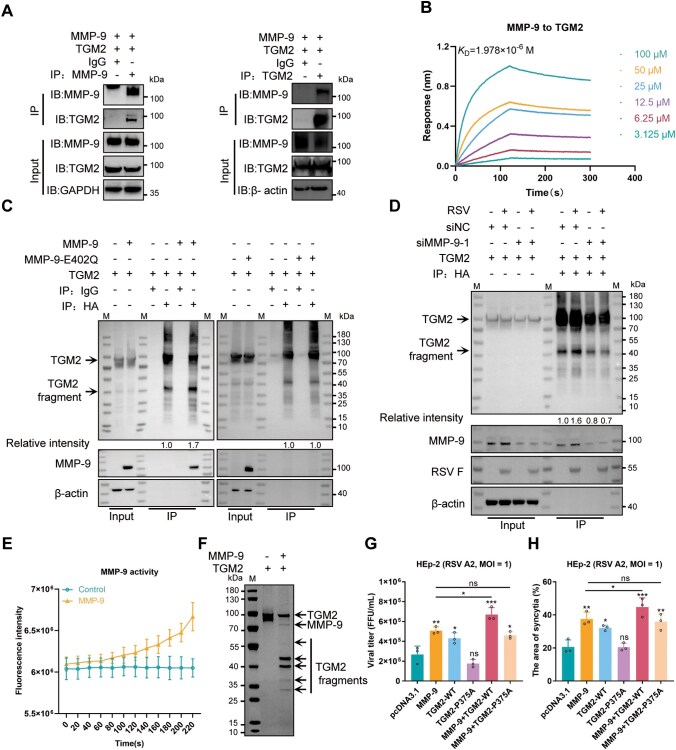
MMP-9 cleaves TGM2 to potentiate RSV infection. (A) MMP-9–TGM2 interaction. Co-immunoprecipitation (Co-IP analysis in HEK293T cells co-transfected with MMP-9 and TGM2 plasmids (48 h). Immunoblots probed with anti-MMP-9, anti-TGM2, anti-GAPDH, or anti-β-actin control. Blots represent three independent experiments. (B) Binding kinetics. Biolayer interferometry (BLI) quantification of MMP-9–TGM2 interaction affinity. The results represent three biological replicates. (C) Catalytic-dependent complex formation. Co-IP of HA-TGM2 with wild-type MMP-9 or catalytically inactive mutant (E402Q) in HEK293T cells (48 h). β-actin as loading control. TGM2 fragment band intensity was quantified by ImageJ software (gray values). Blots represent three independent experiments. (D) Infection-enhanced cleavage. MMP-9-dependent TGM2 proteolysis in HEp-2 cells transfected with HA-TGM2 and siMMP-9, followed by RSV A2 infection (MOI = 1, 72 hpi). TGM2 fragment band intensity was quantified by ImageJ software (gray values). Blots represent three independent experiments. (E) Enzymatic validation. MMP-9 proteolytic activity was measured using fluorogenic substrate Mca-PLGL-Dpa-AR-NH2. The results represent three biological replicates. (F) *In vitro* cleavage assay. Recombinant TGM2 (2 µg) was incubated with APMA-activated MMP-9 (0.2 µg) for 18 h (37°C, pH 7.5). Cleavage products resolved by SDS-PAGE (10%). Arrows indicate proteolytic fragments. The results represent three biological replicates. (G) Functional rescue. Viral titers (FFA) in HEp-2 cells overexpressing MMP-9, TGM2, or cleavage-resistant mutant (TGM2-P375A) post RSV infection (MOI = 1, 72 hpi). mean ± SD, *n* = 3. (H) Syncytium rescue. Syncytia area quantification in MMP-9/TGM2-modulated cells (ImageJ software). mean ± SD, *n* = 3. The results are representatively shown with three random experiments. The error bars indicate the mean ± SD of three technical replicates (*n* = 3). Statistical analysis: one-way ANOVA with Dunnett’s post hoc test (G and H); unpaired two-tailed Student’s *t*-test (G and H). *P < 0.05, **P < 0.01, ***P < 0.001; ns, not significant.

To investigate the interaction and functional relationship between MMP-9 and TGM2, we first confirmed their exogenous interaction in HEK293T cells through a Co-IP assay (Fig. 6A). This interaction was further validated by a BLI assay, which demonstrated a physical association between purified human MMP-9 and TGM2 proteins with a dissociation constant (*K*_D_) of 1.978 × 10^−6^ mol/L (Fig. 6B), indicating a specific and direct interaction.

To examine whether MMP-9 cleaves TGM2, we constructed a N-terminal HA-tagged TGM2 cDNA and co-expressed it with MMP-9 in HEK293T cells. Co-IP assays revealed that TGM2 was cleaved, producing a 44 kDa fragment alongside the full-length TGM2 (Fig. 6C, left). Exogenous expression of MMP-9 markedly enhanced the intensity of the 44 kDa band. This cleavage was dependent on MMP-9’s protease activity, as the enzymatically inactive mutant MMP-9 E402Q failed to enhance cleavage or generate the 44 kDa fragment (Fig. 6C, right). In HEp-2 cells, RSV infection significantly increased TGM2 cleavage, which was abolished upon MMP-9 knockdown (Fig. 6D), confirming MMP-9’s role in TGM2 processing during RSV infection.

*In vitro* cleavage assays using purified MMP-9 and TGM2 proteins further confirmed MMP-9’s ability to cleave TGM2. Three major bands were observed: 100 kDa (full-length TGM2), 44 kDa, and 41 kDa (Fig. 6E and 6F). N-terminal sequencing of the 41 kDa fragment showed the N-terminal sequence is VRAIK, demonstrating the cleavage site at PVP^375^-VR. Consequently, the 44 kDa (TGM2-44) band corresponded to the N-terminal fragment cleaved at V375, whereas the 41 kDa (TGM2-41) band matched the C-terminal fragment. Additional faint bands suggested potential secondary cleavage sites.

To validate the function of the Pro-375 cleavage site, we expressed wild-type TGM2 (TGM2-WT) and a cleavage-resistant mutant (TGM2-P375A) in HEp-2 cells. TGM2-WT, but not TGM2-P375A, enhanced viral replication and syncytium formation. Co-expression of MMP-9 with TGM2-WT further amplified these effects, whereas TGM2-P375A failed to produce any significant enhancement (Fig. 6G and 6H). These results demonstrate that MMP-9 cleaves TGM2 at Pro-375, a modification critical for TGM2’s role in promoting RSV infection and syncytium formation.

### The PDI activity of the TGM2 N terminus promotes RSV fusion protein-mediated syncytia formation

**Figure 7.**
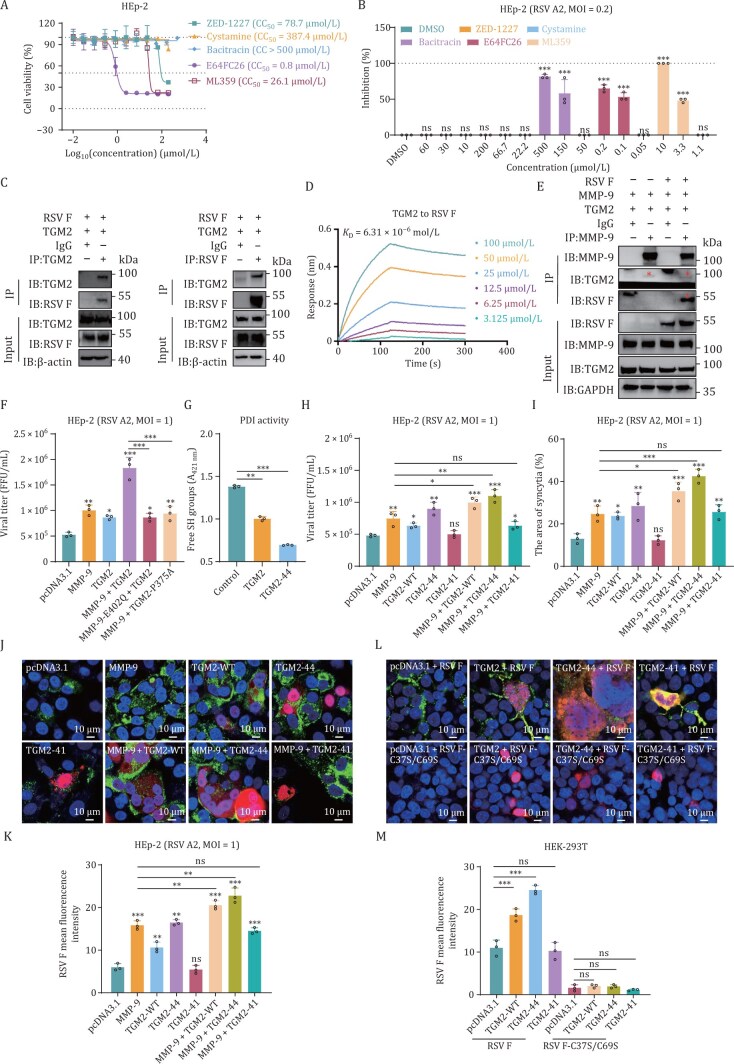
MMP-9-mediated TGM2 cleavage potentiates RSV–host interaction. (A) Cytotoxicity profiling. Dose-response curves of TGM2 inhibitors (ZED-1227, Cystamine, Bacitracin, E64FC26, ML359) in HEp-2 cells (72 h treatment). Viability was assessed by CellTiter-Glo assay. Data normalized to untreated controls. mean ± SD, *n* = 3. (B) Antiviral efficacy. Viral inhibition (FFA) in RSV A2-infected HEp-2 cells treated with TGM2 inhibitors at threefold dilution gradients: ZED-1227 (60–10 µmol/L), Cystamine (200–22.2 µmol/L), Bacitracin (500–50 µmol/L), E64FC26 (0.2–0.05 µmol/L), and ML359 (10–1.1 µmol/L). DMSO as vehicle control. mean ± SD, *n* = 3. (C) TGM2–F protein interaction. Co-IP analysis in HEK293T cells co-expressing TGM2 and RSV F (48 h). Immunoblots were probed with anti-TGM2, anti-F, or IgG control. Blots represent three independent experiments. (D) Binding kinetics. BLI quantification of TGM2–DS-Cav1 (RSV F prefusion conformation) interaction. The results represent three biological replicates. (E) Ternary complex formation. Co-IP of MMP-9-TGM2-F complex in HEK293T cells (48 h). GAPDH as loading control. Below the asterisk represents the objective band. Blots represent three independent experiments. (F) Impairing MMP-9/TGM2/RSV-F complex inhibits RSV infection. Viral titers (FFA) in HEp-2 cells expressing wild-type, mutant variants of MMP-9 (catalytically inactive MMP-9-E402Q) and TGM2, mutant variants of TGM2 (cleavage site-inactive TGM2-P375A) in various combinations post RSV infection (MOI = 1, 72 hpi). mean ± SD, *n* = 3. (G) Proteolytic enhancement. MMP-9-cleaved TGM2 (TGM2-44) enhances protein disulfide isomerase (PDI) activity was measured using reduced DS-Cav1. mean ± SD, *n* = 3. (H) Truncation functional analysis. Viral titers (FFA) in HEp-2 cells expressing MMP-9, TGM2, or cleavage fragments (TGM2-44/41) post RSV infection (MOI = 1, 72 hpi). mean ± SD, *n* = 3. (I) Syncytium rescue. Syncytia area quantification in TGM2 truncation-expressing cells (ImageJ software). mean ± SD, *n* = 3. (J) Subcellular redistribution. Confocal microscopy of HEp-2 cells co-expressing TGM2 truncations (HA/FLAG-tagged) and RSV F (72 hpi). Scale bar: 10 µm. Images represent three biological replicates. (K) F protein expression. Fluorescence intensity quantification of RSV F in (J) (ImageJ software). mean ± SD, *n* = 3. (L) Fusion competence assay. Syncytium formation in HEK293T cells co-expressing TGM2 truncations and RSV F/F-C37S/C69S mutants (48 h). Scale bar: 10 µm. Images represent three biological replicates. (M) Mutant validation. RSV F expression levels in (L) were quantified by ImageJ software. mean ± SD, *n* = 3. The results are representatively shown with three random experiments. The error bars indicate the mean ± SD of three technical replicates (*n* = 3). Statistical analysis: one-way ANOVA with Dunnett’s post hoc test (B, F, G, H, I, K, M); unpaired two-tailed Student’s *t*-test (F, H, I, K, M). *P < 0.05, **P < 0.01, ***P < 0.001; ns, not significant.

TGM2 is a multifunctional enzyme with established transamidation, GTP/ATPase, and PDI activities (Kanchan et al., 2015). To identify enzymatic activities of TGM2 associated with RSV infection, we screened inhibitors targeting functions of enzyme: ZED-1227 (transamidation inhibitor), Cystamine (GTP/ATPase inhibitor), and Bacitracin, E64FC26, or ML359 (PDI inhibitors). Cytotoxicity assays confirmed safe concentrations for each inhibitor (Fig. 7A). ZED-1227 and Cystamine did not inhibit RSV replication. All PDI inhibitors Bacitracin, E64FC26, or ML359 exhibited strong antiviral efficacy (Fig. 7B), indicating TGM2’s PDI activity is involved in RSV infection. Subsequently, Co-IP assay revealed the interaction between TGM2 and RSV F protein (Fig. 7C). Binding kinetics analysis via BLI further confirmed this interaction, demonstrating moderate affinity (*K*_D_ = 6.31 × 10^−6^ mol/L) between TGM2 and RSV F protein (Fig. 7D). Furthermore, co-expressing MMP-9, TGM2, and RSV F revealed ternary complex formation (Fig. 7E). Functional disruption of MMP-9 or TGM2 impaired RSV infectivity (Fig. 7F), demonstrating coordinated regulation. We hypothesize that MMP-9-mediated cleavage of TGM2 enhances its PDI activity, facilitating disulfide bond formation in RSV F to promote fusion-competent trimer maturation. Indeed, the cleavage of TGM2 by activated MMP-9 resulted in a ~1.3-fold increase in PDI activity (Fig. 7G), though basal activity remained low.

To further dissect the functional contributions of MMP-9-generated TGM2 fragments (TGM2-44 [N-terminal] and TGM2-41 [C-terminal]), we analyzed their effects on RSV infection. Overexpression of TGM2-WT or TGM2-44, but not TGM2-41, enhanced viral production and syncytium formation in HEp-2 cells (Fig. 7H and 7I). Consistent with these findings, surface staining revealed elevated F-protein levels on cells overexpressing MMP-9, TGM2-WT, or TGM2-44, but not in controls (Fig. 7J and 7K). To confirm the role of disulfide bond formation, we introduced cysteine-to-serine mutations (C37S/C69S) into RSV F. Unlike wild-type F, the C37S/C69S mutant failed to exhibit enhanced membrane trafficking or syncytium formation in the presence of TGM2 or its N-terminal fragment (Fig. 7L and 7M).

These data show that MMP-9 cleavage of TGM2 generates an N-terminal fragment (1–375 aa) with augmented PDI activity, which catalyzes disulfide bond formation in RSV F to promote trimer maturation, membrane trafficking, and syncytium formation.

### MMP-9 is a critical host factor promoting RSV pathogenesis

**Figure 8.**
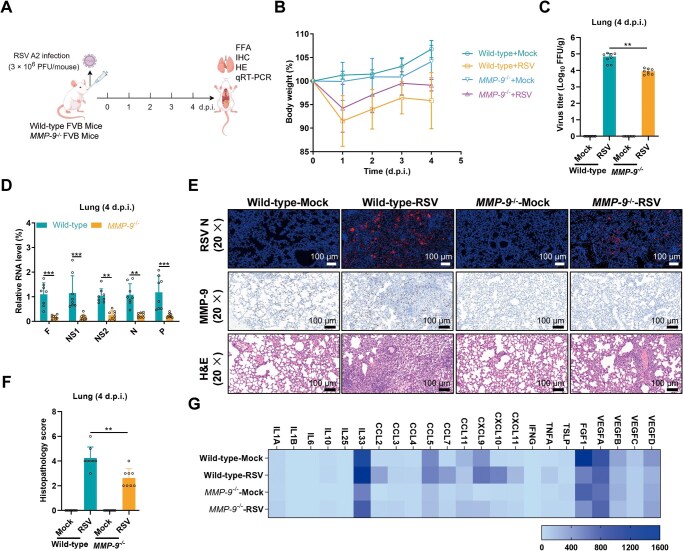
MMP-9 drives RSV pathogenesis in a murine model. (A) Experimental timeline. Wild-type (WT) and MMP-9 knockout (*MMP-9* ^−/−^) mice were intranasally infected with RSV A2 (3 × 10^6^ PFU/mouse) and euthanized at 4 days post-infection (dpi). (B) Clinical progression. Percent body weight change in RSV-infected mice relative to baseline. mean ± SD, *n* = 8/group. (C) Viral burden reduction. Lung viral loads quantified by FFA at 4 dpi. mean ± SD, *n* = 8/group. (D) Viral transcriptome. RSV gene expression (F, NS1, NS2, N, P) in lungs normalized to β-actin (4 dpi). mean ± SD, *n* = 8/group. (E) Histopathological analysis. Lung sections from infected mice stained for RSV N protein (immunofluorescence, top), MMP-9 protein (immunohistochemistry, middle), and inflammatory infiltration (H&E, bottom). Scale bar: 100 µm. Images represent three biological replicates. (F) Quantitative scoring of the lung pathology from mice at 4 dpi. mean ± SD, *n* = 8/group. (G) Cytokine dysregulation. mRNA levels of proinflammatory mediators in lung homogenates (4 dpi). The results are representatively shown with three random experiments. The error bars indicate the mean ± SD of eight technical replicates (*n* = 8). Statistical significance was determined by unpaired two-tailed Student’s *t*-test (C, D, F): **P < 0.01, ***P < 0.001.

To evaluate the role of MMP-9 in RSV infection, we infected WT and MMP-9 knockout (*MMP-9* ^−/−^) mice (Fig. 8A). WT mice exhibited significantly greater weight loss than *MMP-9* ^−/−^ mice throughout the infection course, with a pronounced divergence at 1-day post-infection (dpi) (Fig. 8B). By 4 dpi, lung viral titers in *MMP-9* ^−/−^ mice were reduced by 0.81 log compared to WT controls (Fig. 8C), and viral gene expression remained elevated in WT lungs relative to *MMP-9* ^−/−^ mice (Fig. 8D).

Immunofluorescence staining of lung tissues revealed viral antigen N protein accumulation in alveolar cells of WT mice at 4 dpi (Fig. 8E, top panel), whereas *MMP-9* ^−/−^ mice showed attenuated viral replication (Fig. 8E, top panel). Immunohistochemical analysis of lung tissues demonstrated that markedly increased MMP-9 expression was observed in WT mice with RSV infection, and MMP-9 expression was undetectable in *MMP-9* ^−/−^ mice regardless of RSV infection (Fig. 8E, middle panel). Histopathological analysis further demonstrated that RSV-infected WT mice developed extensive lung lesions and pronounced inflammatory cell infiltration, whereas *MMP-9* ^−/−^ mice exhibited milder pathology (Fig. 8E, bottom panel and quantified in Fig. 8F). Consistent with these findings, RSV infection triggered a broad upregulation of proinflammatory cytokines and chemokines in WT lungs, including IL-1β, IL-6, TNF-α, IFN-γ, and CXCL10, among others (Andrade et al., 2020). In contrast, *MMP-9* ^−/−^ mice displayed significantly reduced levels of these factors (Fig. 8G). These data show that MMP-9 as a critical host factor exacerbating RSV pathogenesis, as MMP-9 deficiency attenuates viral replication, tissue damage, and inflammatory responses.

### Metalloproteinase inhibitors attenuate RSV infection and pathology *in vivo*

**Figure 9.**
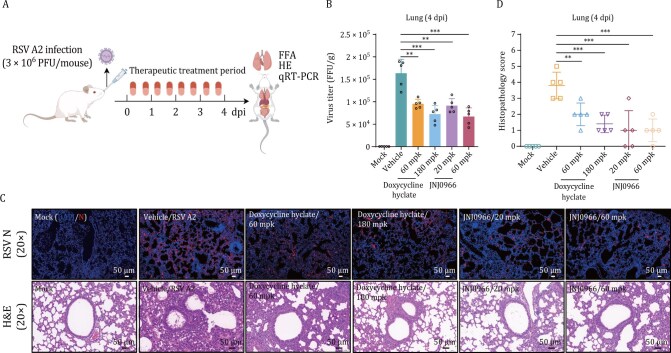
Therapeutic efficacy of MMP-9 inhibitors against RSV in mice. (A) Therapeutic regimen. BALB/c mice received oral gavage of doxycycline hyclate (60/180 mg/kg) or JNJ0966 (20/60 mg/kg) twice daily (b.i.d.) starting 2 hours post RSV A2 infection (3 × 10^6^ PFU/mouse, *n* = 5/group). Controls: HP-β-CD (vehicle) and uninfected mice. (B) Viral load reduction. Lung viral titers quantified by FFA at 4 dpi. mean ± SD, *n* = 5/group. (C) Pathological evaluation. Top: Immunofluorescence detection of RSV N protein in lung sections. Bottom: H&E staining showing inflammatory infiltration. Scale bars: 50 µm. Images represent two biological replicates. (D) Histopathology scoring. Blinded assessment of pulmonary inflammation (0–4 scale: 0 = normal, 4 = severe confluent pathology). Data pooled from two independent experiments. mean ± SD, *n* = 5/group. The results are representatively shown with two random experiments. The error bars indicate the mean ± SD of five technical replicates (*n* = 5). Statistical significance was determined by one-way ANOVA with Dunnett’s post hoc test (B, D): **P < 0.01, ***P < 0.001.

To evaluate the antiviral efficacy of metalloproteinase inhibitors, we employed a BALB/c mouse model intranasally challenged with 3 × 10^6^ PFU RSV A2. Mice received twice-daily oral administration of JNJ0966 (selective targeting of MMP-9 (Scannevin et al., 2017), 20 or 60 mg/kg [mpk]) or doxycycline hyclate (a broad-spectrum MMP inhibitor (Patnaik et al., 2021; Samtani et al., 2009), 60 or 180 mpk), beginning 2 hpi (Fig. 9A). By 4 dpi, JNJ0966 (60 mpk) and doxycycline hyclate (180 mpk) reduced lung viral titers by 2.6-fold and 2.0-fold, respectively, compared to vehicle-treated controls (Fig. 9B). Consistent with these findings, immunofluorescence analysis revealed abundant viral antigen in lungs of vehicle-treated mice, whereas both inhibitors substantially suppressed nucleocapsid protein expression (Fig. 9C, top panel). Histopathological assessment further demonstrated that vehicle-treated mice exhibited severe alveolar epithelial hyperplasia, alveolar wall thickening, and inflammatory infiltration. In contrast, inhibitor-treated mice showed minimal lung pathology, with attenuated alveolar damage and inflammation (Fig. 9C, bottom panel, and quantified in Fig. 9D). These results show that metalloproteinase inhibitors, particularly JNJ0966, effectively curb RSV replication and mitigate infection-associated lung injury.

## Discussion

**Figure 10.**
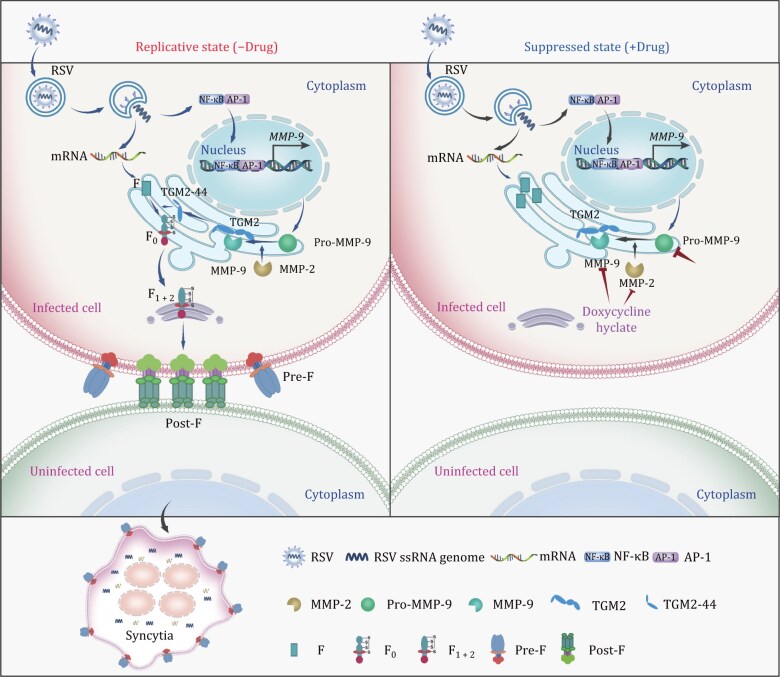
Model for the function of MMP-9 in RSV infection. RSV particles enter the cell through membrane fusion or endocytosis by interacting with host receptors. Following entry, the release and uncoating of the incoming genomic RNA subject it to RNA replication, transcription, and translation with the help of structural proteins. The induction of MMP-9 expression is mediated through NF-κB or AP-1 signaling pathways. MMP-9 cleaves TGM2 and enhances PDI activity, which leads to RSV fusion protein transport to the host cell surface and promotes RSV fusion protein-mediated syncytia formation. This process can be inhibited by JNJ0966, a selective inhibitor of MMP-9, or doxycycline hyclate, a broad-spectrum MMP inhibitor.

As previously reported, the participation of host protease in viral functions is widespread. Our study uncovers a previously unrecognized mechanism by which MMP-9 promotes RSV infection through proteolytic activation of TGM2. While MMP-9 is known to facilitate diverse viral infections from HBV immune evasion to SARS-CoV-2 spike priming, its role in RSV pathogenesis has remained mechanistically unclear. Here, we demonstrate that MMP-9 facilitates RSV replication by enhancing TGM2’s PDI activity, enabling proper folding of the RSV F protein into fusion-competent trimers. This contrasts with the canonical roles of MMP-9 in extracellular matrix degradation or cytokine processing, highlighting a unique virus–host interface where protease activity is co-opted for viral structural maturation (Fig. 10).

MMP-9 exhibits context-dependent pleiotropic effects in viral infections, as demonstrated in HBV (Chen et al., 2017; Yang et al., 2019), ZIKA (Hui et al., 2020), and SARS-CoV-2 (Benlarbi et al., 2022). MMP-9 facilitates HBV replication through repressing IFN/JAK/STAT signaling, MMP-9 facilitates ZIKA invasion of the testis by modulating the integrity of the blood-testis barrier, and may facilitate SARS-CoV-2 cellular entry via spike protein cleavage and membrane fusion promotion, thereby serving as a critical host factor for viral infection. Our findings reveal a distinct paradigm for RSV, where MMP-9 operates not as a direct viral protease but as a molecular activator of host-derived TGM2. Structural analysis of TGM2 reveals that its N-terminal β-sandwich domain and catalytic core domain (1–375 aa) harbor the PDI-active site (Stamnaes et al., 2010), while its C-terminal domain regulates substrate binding and cellular localization (Singh et al., 2016). MMP-9 cleavage at the interdomain linker liberates the N-terminal fragment, releasing its latent PDI activity (Fig. 7G). This fragment catalyzes disulfide bond formation in RSV F (C37–C439/C69–C212), stabilizing the prefusion trimer and enabling membrane fusion, which mediates RSV cell-to-cell infection. Notably, this mechanism bypasses the need for MMP-9 to directly process RSV F, resolving prior controversies about its role in RSV proteolysis (Xu et al., 2021). This might also be the reason why MMP-9 does not function at the early stage of RSV infection (Fig. 2).

MMP-9 induction during RSV infection creates a feedforward loop: viral replication triggers inflammatory signaling (e.g., IL-1, TNF-α) that upregulates MMP-9 (Figs. S5 and S6) (Yan and Boyd, 2007), which in turn enhances viral spread via TGM2 activation. This duality amplifies both viral fitness and immunopathology, positioning MMP-9 as a central coordinator of RSV virulence. Our observation that MMP-9 deficiency attenuates both viral titers and cytokine storms (Fig. 8C and 8G) suggests that therapeutic targeting of MMP-9 could simultaneously curb viral replication and mitigate tissue damage. We propose that MMP-9’s dual roles may reflect evolutionary adaptation by RSV to exploit host inflammatory responses for dissemination—a strategy potentially shared by other pneumotropic viruses.

The superior efficacy of JNJ0966 (a selective MMP-9 inhibitor) over broad-spectrum agents like doxycycline hyclate underscores the importance of target specificity. While doxycycline hyclate’s anti-RSV activity likely arises from collateral MMP inhibition, JNJ0966’s precision in blocking the MMP-9/TGM2 axis minimizes off-target effects on homeostatic proteolysis. This selectivity is critical, as complete MMP-9 ablation may impair tissue repair mechanisms. Our data suggest that timed inhibition of MMP-9 during acute infection, rather than chronic suppression, could balance antiviral efficacy with host recovery.

There are some unresolved questions. First, while MMP-2 knockdown reduces RSV replication (Fig. 1H) and interacts with MMP-9 (Fig. 5A and 5B), the exact regulatory mechanism remains unclear. Based on established literature demonstrating MMP-2-mediated activation of pro-MMP-9 (Li et al., 2017; Mook et al., 2004; Toth et al., 2003), we propose that MMP-2 processes pro-MMP-9 to its active form, which then cleaves TGM2 to enhance PDI activity—creating an MMP-2-MMP-9-TGM2-PDI cascade that promotes RSV replication. While we focused on the N-terminal PDI domain, the C-terminal fragment (376–687 aa) generated by MMP-9 cleavage lacks autonomous activity in our assays. We hypothesize that it may regulate full-length TGM2 localization or substrate accessibility, creating an autoregulatory circuit. Our study demonstrates TGM2’s direct role in RSV fusion, yet its essential physiological functions preclude its viability as a therapeutic target. TGM2 knockout induces lethality in most murine strains (De Laurenzi and Melino, 2001), and even in permissive C57BL/6 mice causes metabolic dysfunction (Bernassola et al., 2002), confounding antiviral studies. While we did not directly assess TGM2’s regulation of viral replication *in vivo*, these constraints strongly support targeting upstream MMP-9 instead. MMP-9 offers clinical advantages through available inhibitors (doxycycline hyclate/JNJ0966), and a broader therapeutic window as an upstream regulator. Future studies should employ TGM2-P375A mutants to specifically probe its enzymatic role without systemic disruption.

This study reveals how RSV replication depends on host protease systems, identifying MMP-9 as the master regulator of viral membrane fusion. The MMP-9/TGM2 interaction presents a promising drug target that could block RSV’s ability to exploit host cell redox systems. This antiviral strategy may extend to other enveloped viruses using similar disulfide-dependent fusion mechanisms.

## Key conclusions and novel interpretation

These findings suggest that MMP-9 serves as a crucial host factor that exacerbates the pathogenesis of RSV, as the deficiency of MMP-9 mitigates viral replication, tissue damage, and inflammatory responses. Notably, the diminished cytokine storm in *MMP-9* ^−/−^ mice suggests that MMP-9 may amplify immunopathology not only by facilitating viral spread but also by potentiating immune hyperactivation—a mechanism warranting further investigation.

## Supplementary data

Supplementary data is available at *Protein & Cell* online https://doi.org/10.1093/procel/pwaf063.

pwaf063_Supplementary_Data

## Data Availability

The data generated in this study are available within the manuscript and its supplementary data files. The complete sequences of RSV A2 (GenBank: KT992094) and RSV B18537 (GenBank: MG813995) are available on GenBank. The RNA-seq raw data are available from the NCBI Sequence Read Archive under BioProject Accession ID PRJNA1247189 and PRJNA1247202.

## Abbreviations

ACE2: angiotensin converting enzyme 2
BLI: biolayer interferometry
COL5A1: collagen type V alpha 1 chain
Co-IP: co-immunoprecipitation
CPE: cytopathic effect
CXCL10: C-X-C motif chemokine ligand 10
DCAF7: DDB1 and CUL4 associated factor 7
dpi: day post-infection
ELISA: enzyme-linked immunosorbent assay
F: fusion glycoprotein
FBXL15: F-Box and leucine rich repeat protein 15
G6PD: glucose-6-phosphate dehydrogenase
hpi: hour post-infection
IFN-γ: interferon gamma
IGHV3-49: immunoglobulin heavy variable 3-49
IL-1β: Interleukin-1 beta
IL-6: interleukin 6
ITIH5: inter-alpha-trypsin inhibitor heavy chain 5
MMP-9: matrix metalloproteinase 9
MMPs: matrix metalloproteinases
MT1-MMP: membrane type 1 matrix metalloproteinase
NCL: nucleolin
PDI: protein disulfide isomerase
RSV: respiratory syncytial virus
SARS-CoV-2: , Severe acute respiratory syndrome coronavirus 2;
TGM2: , transglutaminase 2
TMPRSS2: , transmembrane serine protease 2
TNF-α: tumor necrosis factor alpha
WT: wild-type
ZIKV: , Zika virus
