# Supplementary material for: MMP-9 regulates disulphide isomerase activity of TGM2 to enhance fusion glycoprotein-mediated syncytium formation of respiratory syncytial virus
Source: Protein Cell. 2025 Aug 11;17(1):59–76. doi: 10.1093/procel/pwaf063 (PMC12888920; doi:10.1093/procel/pwaf063)
Supplement: pwaf063_Supplementary_Data [file pwaf063_supplementary_data.pdf]

Supplemental information

MMP-9 regulates disulphide isomerase activity of TGM2 to enhance fusion glycoprotein-mediated syncytium formation of respiratory syncytial virus

Bao Xue, Anqi Zhou, Yihang Zhong, Yuhan Mao, Ran Peng, Yuhang Chen, Jiayi Zhong, Junjun Liu, Yuan Zhou, Yuying Fang, Wei Zhang, Jielin Tang, Wei Peng, Jia Liu, Qi Yang, and Xinwen Chen

This file includes:

- **Figures S1-7**
- **Tables S1-3**

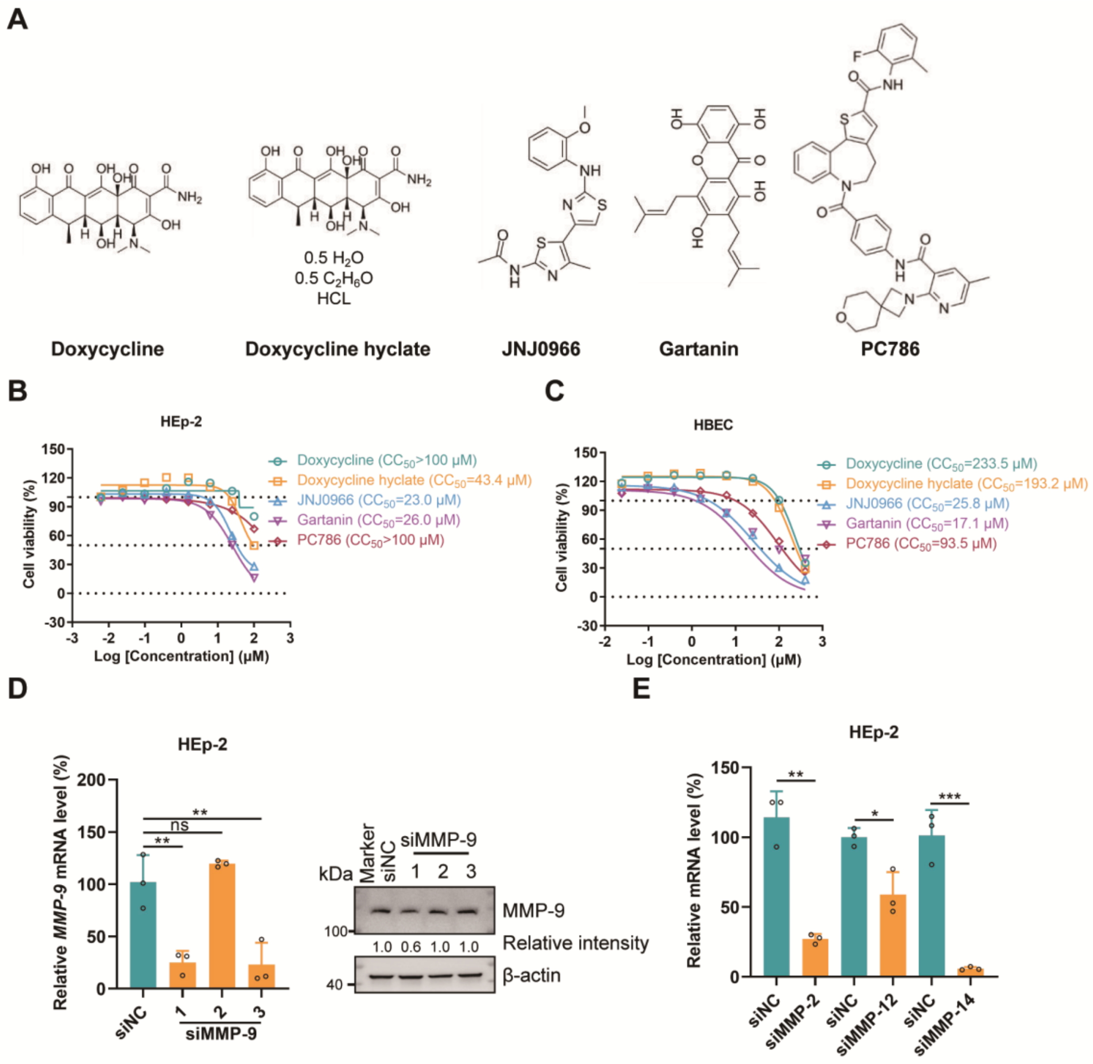

Figure S1. Compound cytotoxicity and MMP knockdown validation

(A) Chemical structures of tested compounds: doxycycline, doxycycline hyclate, JNJ0966, gartanin, and PC786.

(B-C) Cytotoxicity profiles in HEp-2 (B) and HBEC (C) cells. Cells were treated with a 4-fold serial dilution (0 − 100/400 μM) for 72 h. Viability was assessed using the CellTiter-Glo assay (G7570, Promega). Dashed lines indicate 50% cytotoxic concentration (CC_{50}) calculated by nonlinear regression (GraphPad Prism 8.0). mean ± SD, n=3.

(D-E) MMP knockdown efficiency in HEp-2 cells transfected with 20 nM siRNAs

targeting MMP-9 (siMMP-9) (D), MMP-2 (siMMP-2), MMP-12 (siMMP-12), or MMP-14 (siMMP-14) (E) for 48 h. mRNA levels quantified by RT-qPCR normalized to siNC (mean ± SD, n=3), MMP-9 protein suppression confirmed by Western blot. Membranes were probed with anti-MMP-9 (#13667, CST) or anti-β-actin (66009-1-Ig, Proteintech). Band intensity quantified by ImageJ software (gray values).

The results are representatively shown with three random experiments. The error bars indicate the mean ± SD of three technical replicates (n=3). Statistical analysis: one-way ANOVA with Dunnett's post hoc test (D); unpaired two-tailed Student's t-test (E). *$p < 0.05$, **$p < 0.01$, ***$p < 0.001$; ns, not significant.

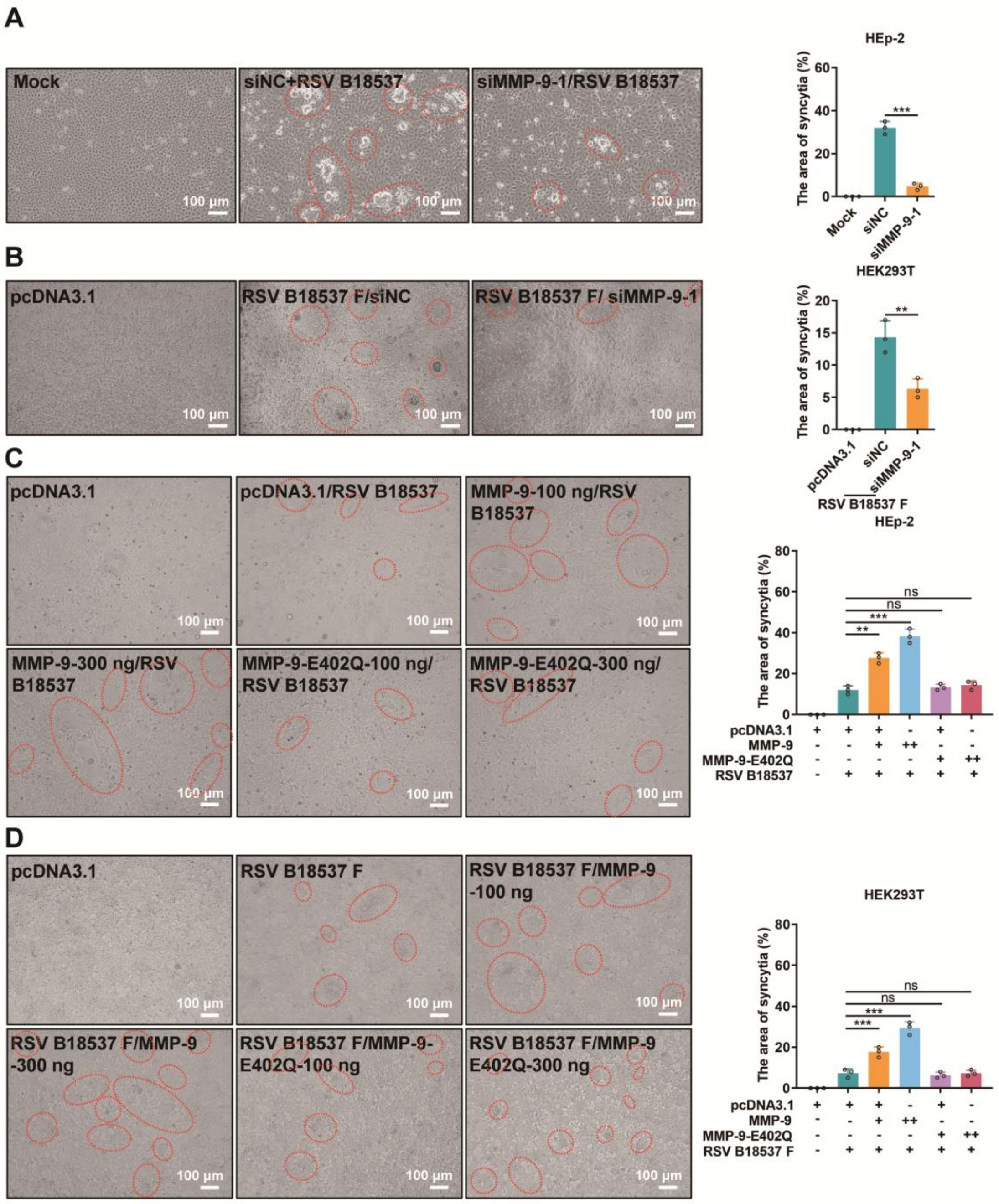

Figure S2. MMP-9 promotes RSV strain-independent syncytium formation

(A) MMP-9 knockdown suppresses RSV B18537-induced syncytia. HEp-2 cells were transfected with 20 nM siMMP-9 for 24 h and then infected with RSV B18537 (MOI=0.2). The syncytia area was quantified by ImageJ software at 72 hours post-infection (hpi). Red circles highlight representative syncytia. mean ± SD, n=3.

(B) MMP-9 knockdown suppresses syncytium formation in HEK-293T cells co-

transfected with siNC/siMMP-9 (20 nM) and RSV B18537 F plasmid (500 ng). Syncytia (red circles) were quantified 48 h post-transfection. mean ± SD, n=3.

(C) Catalytic activity-dependent enhancement of syncytogenesis. HEp-2 cells were transfected with wild-type MMP-9 or catalytically inactive mutant (E402Q) at 100/300 ng, followed by RSV B18537 infection (MOI=0.2, 72 hpi). The syncytia area was normalized to the empty vector control. mean ± SD, n=3.

(D) MMP-9-driven syncytogenesis in HEK293T cells. HEK293T cells were co-transfected with MMP-9/E402Q (100/300 ng) and RSV B18537 F plasmid (500 ng) for 48 h. Syncytia formation in cells. The syncytia area was normalized to the empty vector control. mean ± SD, n=3.

Images represent three biological replicates comprising nine fields each. The error bars indicate the mean ± SD of three biological replicates (n=3). Statistical significance was determined by unpaired two-tailed Student's t-test (A, B); one-way ANOVA with Dunnett's post-test (C, D). $^{**}p < 0.01$, $^{***}p < 0.001$; ns, not significant. Scale bar: 100 µm.

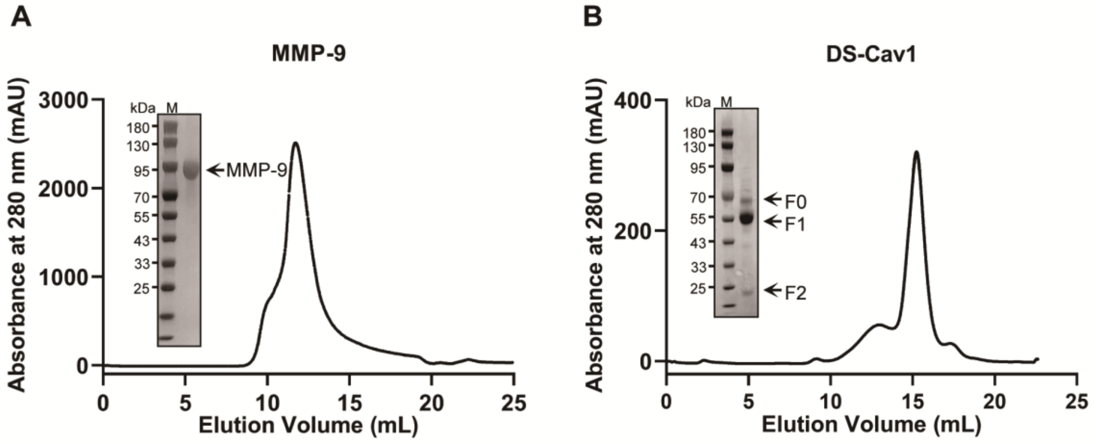

Figure S3. Characterization of purified MMP-9 and DS-Cav1

(A) Recombinant MMP-9 purification. Right: Size-exclusion chromatography (SEC) elution profile of His-tagged human MMP-9 on Superose 6 10/300 GL gel filtration column (17517201, Cytiva). Buffer: PBS. Left: Coomassie Brilliant Blue-stained SDS-PAGE (4-20% gradient gel) of purified MMP-9. Lane 1: Molecular weight markers (kDa); Lane 2: purified MMP-9 (predicted MW: 92 kDa).

(B) Proteolytic processing of DS-Cav1 prefusion F protein. Right: SEC profile of papain-digested DS-Cav1 fragment on Superose 6 10/300 GL gel filtration column (17517201, Cytiva). Left: Coomassie-stained SDS-PAGE analysis of digestion products. Lane 1: Molecular weight markers (kDa); Lane 2: Digested fragments (predicted MW: 67 kDa F0, 56 kDa F1, 21 kDa F2).

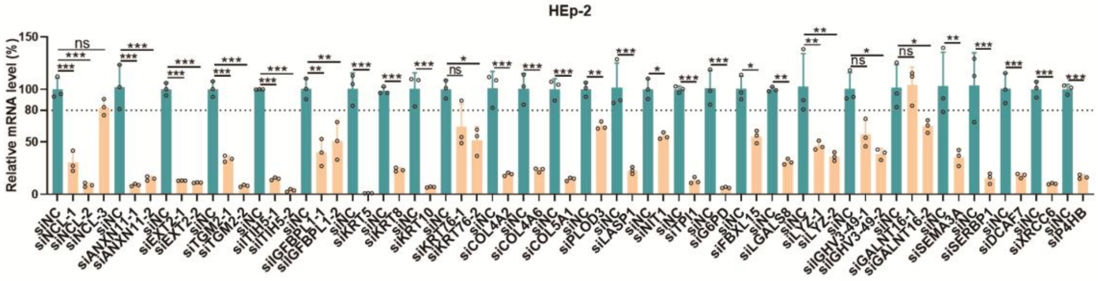

Figure S4. siRNA screening identifies host factors regulating RSV syncytia

Validation of siRNA-mediated gene Silencing efficiency. HEp-2 cells were transfected with 20 nM siRNAs targeting 28 candidate genes for 48 h. RT-qPCR quantified mRNA levels with primers listed in **Table S3.** Data normalized to non-targeting control siRNA (siNC) and β-actin reference gene. The dashed line indicates the 80% silencing threshold. mean ± SD, n=3.

The results are representatively shown with three random experiments. The error bars indicate the mean ± SD of three technical replicates (n=3). Statistical significance was determined by one-way ANOVA with Dunnett's post hoc test (multi-group comparison) and unpaired two-tailed Student's t-test (siRNA vs. siNC): *$p < 0.05$, **$p < 0.01$, ***$p < 0.001$; ns, not significant.

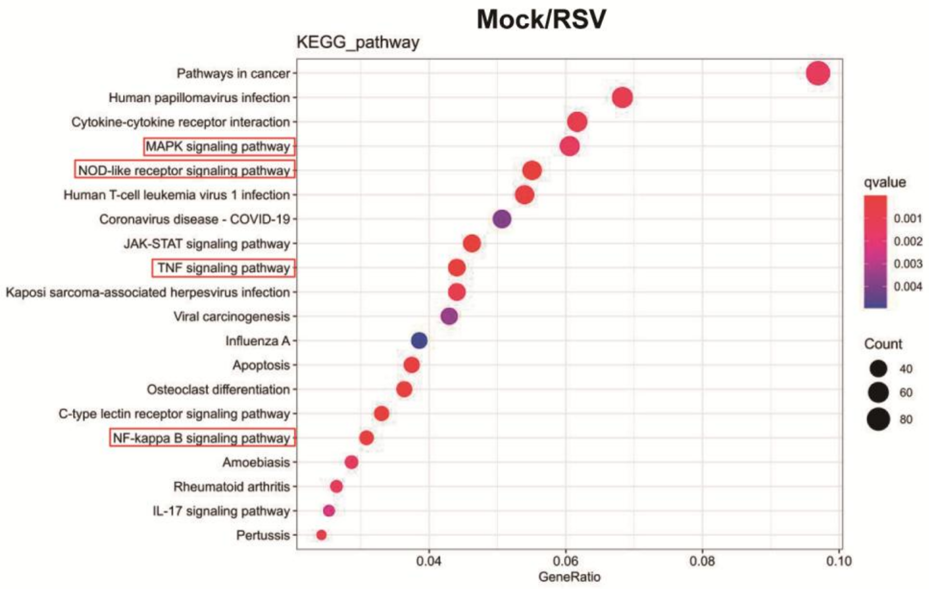

Figure S5. Transcriptomic profiling reveals RSV-activated signaling pathways.

KEGG pathway enrichment analysis of RSV-responsive genes. HEp-2 cells infected with the RSV A2 strain (MOI=1 for 72 hpi) or mock were subjected to RNA sequencing. RNA-seq libraries prepared with NEBNext® Ultra™ II RNA Library Prep Kit for Illumina® (E7770S, NEB), sequenced on Illumina platform (Beijing Tsingke Biotech Co., Ltd.). Differentially expressed genes (DEGs) were defined as $|\log_2 FC| > 1$ with FDR < 0.05 (DESeq2 R package (1.26.0)). Pathway analysis via KOBAS software.

X-axis: Gene ratio (DEGs in pathway / total pathway genes)

Y-axis: Kyoto Encyclopedia of Genes and Genomes (KEGG) pathway

Bubble size: Number of differentially expressed genes per pathway

Color gradient: Adjusted P value (red: most significant)

Data derived from three biological replicates.

Data availability:

• Raw FASTQ files deposited in the NCBI Sequence Read Archive under BioProject

Accession ID PRJNA1247189.

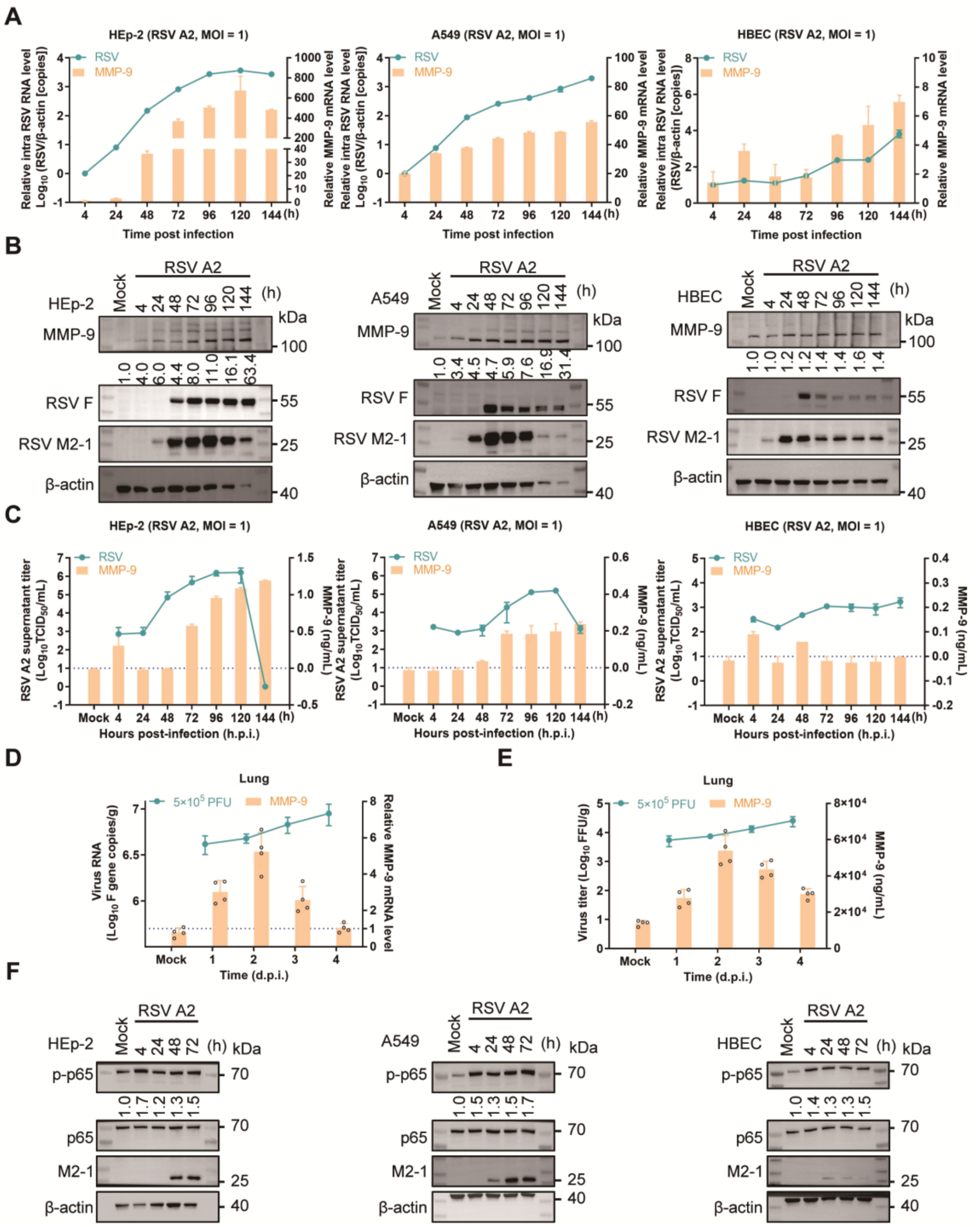

Figure S6. Regulation of MMP-9 during RSV infection

(A-C) *In vitro* dynamics.

(A) Viral replication-MMP-9 transcriptional coupling. HEp-2, A549, and HBEC cells were infected with RSV A2 (MOI=1). Left: RSV F gene RNA quantified by RT-qPCR. Right: MMP-9 mRNA, Timepoints: 4-144 hpi.

(B) Protein-level correlation. Western blot analysis of RSV F (ab43812, Abcam), M2-1 (ab94805, Abcam), and MMP-9 (#13667, CST). β-actin as loading control.

(C) Secretory kinetics. Viral titers ($TCID_{50}$) on HEp-2 cells. and MMP-9 (ELISA with Human MMP9 Quantikine ELISA Kit; DMP900, R&D Systems).

(D-E) *In vivo* kinetics.

(D-E) Active RSV infection: BALB/c mice intranasally were infected with RSV A2 (5×10^5 PFU, n=16/group). (D) Lung viral RNA and MMP-9 mRNA (RT-qPCR, 1-4 dpi). (E) Lung viral load (FFA) and MMP-9 protein (ELISA). mean ± SD, n=4.

(F) Viral replication-NF-κB signaling pathway activation. HEp-2, A549, and HBEC cells were infected with RSV A2 (MOI=1). Western blot analysis of Phospho-NF-κB p65 (#3033, CST), NF-κB p65 (sc-109, Santa Cruz), and M2-1 (ab94805, Abcam), β-actin as loading control. Timepoints: 4-72 hpi.

The results are representatively shown with two random experiments. The error bars indicate the mean ± SD of four technical replicates (n=4).

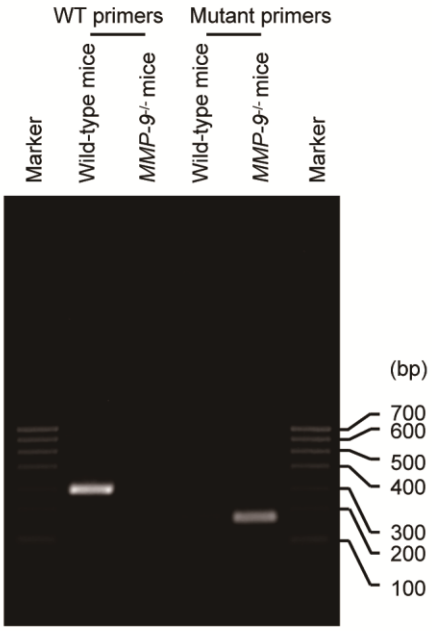

Figure S7. Genotype of MMP-9 knockout

Genotype analysis of wild-type and *MMP-9*$^{-/-}$ mice, PCR analysis of MMP-9, PCR was carried out using two pairs of primers (**Table S3**), PCR product 277 bp (WT) or 172 bp (Mutant).

Table S1. Oligonucleotide primers were used in this study.

Primer names	Sequence (5'-3')
MMP-9 E402Q fw	CGTGGCGGCGCATCAGTTCGGCCACG
MMP-9 E402Q rev	CGTGGCCGAACTGATGCGCCGCCACG
HA-TGM2-FLAG fw	ATATCTGCAGAATTCGCCACCATGTACCCATACGACGTACCAGATTACGCTATGGCCGAGGAGCTGGTCTT
HA-TGM2-FLAG rev	TTTAAACTTAAGCTTGGTACCTTACTTGTCATCGTCGTCCTTGTAATCGCTTCCGGCGGGGCCAATGAT
HA-TGM2-FLAG-P375A fw	TGTGGCCCAGTTGCAGTTCGTGCCATC
HA-TGM2-FLAG-P375A rev	GATGGCACGAACTGCAACTGGGCCACA
HA-TGM2-1-375 fw	ATATCTGCAGAATTCGCCACCATGTACCCATACGACGTACCAGATTACGCTATGGCCGAGGAGCTGGTCTT
HA-TGM2-1-375 rev	CGGGGTACCTCATGGAACTGGGCCACAGCAGTACGTCC
TGM2-376-687-FLAG fw	CGGAATTCGCCACCATGGTTCGTGCCATCAAGGAGGGCGAC
TGM2-376-687-FLAG rev	TTTAAACTTAAGCTTGGTACCTTACTTGTCATCGTCGTCCTTGTAATCGCTTCCGGCGGGGCCAATGAT

Table S2. siRNA sequences were used in this study.

oligonucleotides	Sense/antisense sequence (5'-3')
siNC	UUCUCCGAACGUGUCACGUTT/ACGUGACACGUUCGGAGAATT
siMMP-2	CUGCAAACAGGACAUUGUATT/UACAAUGUCCUGUUUGCAGTT
siMMP-9-1	CUAUGGUCCUCGCCCUGAATT/UUCAGGGCGAGGACCAUAGTT
siMMP-9-2	CGCUCAUGUACCCUAUGUATT/UACAUAGGGUACAUGAGCGTT
siMMP-9-3	GCAUAAGGACGACGUGAAUTT/AUUCACGUCGUCCUUAUGCTT
siMMP-12	CCCGAUGUCCAUCAUUUCATT/UGAAAUGAUGGACAUCGGGTT
siMMP14	GGUCUCAAAUGGCAACAUATT/UAUGUUGCCAUUUGAGACCTT
siNCL-1	CGGCUUUCAAUCUCUUUGUTT/ACAAAGAGAUUGAAAGCCGTT
siNCL-2	CGGCUUUCAAUCUCUUUGUTT/UAACUGUCUUCUUGGCAGGTT
siNCL-3	GGCGAUCUAUUUCCCUGUATT/UACAGGGAAAUAGAUCGCCTT
siIGFBPL1-1	GCGAGUUCGCUCCUGUGGUTT/ACCACAGGAGCGAACUCGCTT
siIGFBPL1-2	GGGUGUGUACCAGUGCCAUTT/AUGGCACUGGUACACACCCTT
siEXT2-1	CCCUCAUCCCAAGAAUGAATT/UUCAUUCUUGGGAUGAGGGTT
siEXT2-2	CCAUCUCCCGGGAGUAUAATT/UUAUACUCCCGGGAGAUGGTT
siANXN11-1	AGGCCAUCAUUGACUGCCUTT/AGGCAGUCAAUGAUGGCCUTT
siANXN11-2	AGGACUAUCUCUCGGGAAUTT/AUUCCCGAGAGAUAGUCCUTT
siTGM2-1	GCUACCAGGGAUCCAGCUUTT/AAGCUGGAUCCCUGGUAGCTT
siTGM2-2	CCAAGUACGAUGCGCCCUUTT/AAGGGCGCAUCGUACUUGGTT
siITIH5-1	GCACUACUUUGCUCCUAAATT/UUUAGGAGCAAAGUAGUGCTT
siITIH5-2	CCAUCAGGCUCCUCAACAATT/UUGUUGAGGAGCCUGAUGGTT
siKRT5	CAUCUCUGUUGUCACAAGCAGUGUU/AACACUGCUUGUGACAACAGAGAUG
siKRT8	GCAUCAGCUCCUCGAGCUUTT/AAGCUCGAGGAGCUGAUGCTT
siKRT10	CUCUGGAGAUAGAACUACATT/UGUAGUUCUAUCUCCAGAGTT
siKRT76-1	GCAGCUAGAUUCACUUCUATT/UAGAAGUGAAUCUAGCUGCTT
siKRT76-2	CUGCCGCAGAGAAUGAGUUTT/AACUCAUUCUCUGCGGCAGTT
siCOL4A2	GACCCAACGGGAUUCCAUUTT/AAUGGAAUCCCGUUGGGUCCC
siCOL4A6	CGACCUGGACCAAUUGGAATT/UUCCAAUUGGUCCAGGUCGTT
siCOL5A1	GAUGGAAUAACAAAGACAACATT/UGUUGUCUUUGUUAUUCCAUCTT
siPLOD3	GGAAGUACAAGGAUGAUGAUGACGACGA/UCGUCGUCAUCAUCAUCCUUGUACUUCC
siLASP1	GGUGAACUGUCUGGAUAAGTT/CUUAUCCAGACAGUUCACCUU
siNIT1	GCUGGAGCAGAGAUACUUATT/UAAGUAUCUCUGCUCCAGCTT
siTPI1	CAAACUGUAUCUUCCUUUATT/UAAAGGAAGAUACAGUUUGGA
siG6PD	ACGAGCUGAUGAAGAGAGUGGGUUU/AAACCCACUCUCUUCAUCAGCUCGU
siFBXL15	GGAACUGCCCAGAACUCCATT/UGGAGUUCUGGGCAGUUCCTT
si LGALS8	CCCACGCCUGAAUAUUAAAGCAUUU/AAAUGCUUUAAUAUUCAGGCGUGGG
siLYZ-1	CCGUCAGUAUGUUCAAGGUTT/ACCUUGAACAUACUGACGGTT
siLYZ-2	GGAGAGUGGUUACAACACATT/UGUGUUGUAACCACUCUCCTT
siIGHV3-49-1	GGGACAUGAGUGGUAGAAATT/UUUCUACCACUCAUGUCCCTT
siIGHV3-49-2	GGGUAGGUUUCAUUAGAAGTT/CUUCUAAUGAAACCUACCCTT
siGALNT16-1	CUCACCUACAUCAGGAAUATT/UAUUCCUGAUGUAGGUGAGTT
siGALNT16-2	GCUUUGAUGAGAAGGCCUATT/UAGGCCUUCUCAUCAAAGCTT
siSEMA3A	UGCAGAAGAUGGACAGUAUTT/AUACUGUCCAUCUUCUGCATT

siSERBP1	GGGUGAAGGAGGCGAAUUUTT/AAAUUCGCCUCCUUCACCCTT
siDCAF7	GGAACAAGCAGGACCCUAATT/UUAGGGUCCUGCUUGUUCCTT
siXRCC6	GAAGUGACAGCUUUGAGAATT/UUCUCAAAGCUGUCACUUCTT
siP4HB	GAUGAACUGUAAUACGCAATT/UUGCGUAUUACAGUUCAUCUU

Table S3. qRT-PCR or PCR primers for gene expression detection and quantification

Gene	Species	Sequence (F/R, 5’-3’)
Fusion protein	RSV	CGAGCCAGAAGAGAACTACCA/CCTTCTAGGTGCAGGACCTTA
NS1	RSV	GGGCAGCAATTCGTTGAGTA/AGCACTGGCATTGTTGTGAA
NS2	RSV	TTGATGAAAGACAGGCCACA/TATCGGCATAGGGAAAGTGC
N	RSV	CTACCCAAGGACATAGCCAACAG/CTTCAACTCTACTGCCACCTCTG
P	RSV	TTTGCTAAGACTCCCCACCGTA/CTTACTACCCAAGGACATAGCCAAC
β-actin	Human	CTCGACACCAGGGCGTTATG/CCACTCCATGCTCGATAGGAT
MMP-2	Human	GACAGTGGATGATGCCTTTGC/GAGCACCAGAGGAAGCCATC
MMP-9	Human	AGACGCCCATTTCGACGATGAC/CAAACCGAGTTGGAACCACGAC
MMP-12	Human	GAACAGCTCTACAAGCCTGGAA/TCTCCAGGTAGATGTGTCCAGT
MMP-14	Human	CATCTGTGACGGGAACTTTGA/GGCAGTGTTGATGGACGCA
NCL	Human	TTGAGGGCAGAGCAATCAGG/AGAGTTTTGGATGGCTGGCT
IGFBPL1	Human	TCGTTCCTCCCCGAAGTGTT/GACAGCTATATTGACATGGTCCC
EXT2	Human	GCCAGCTTGTAACACATCGC/AGGACCTAGAAGCCCTCCAG
ANXN11	Human	CCCACCTGGTAGCAGTTTTC/GGCTGGGGTATTCTTGAGAC
TGM2	Human	ACAAATCCATCAACCGTTCC/GCCAGTTTGTTCAGGTGGTT
ITIH5	Human	TCACCGTGTGCTTCAACATT/GGGTGCCCCAATTAACTCTC
KRT5	Human	AGAAGCCGAGTCCTGGTATCAGAC/CTTGGTGTTGCGGAGGTCATCG
KRT8	Human	CAGAAGTCCTACAAGGTGTCCACTCTGGTTGACCGTAACTGCG
KRT10	Human	GTCCCAACTGGCCTTGAAAC/AGAGCGGATATCTGGGCCT
KRT76	Human	GGAGCCTTGTTTTGAATCCTACA/GGCAGTGCGTTTGTTGATTTC
COL4A2	Human	AGGGTCGCAGGGAGAGCTGG/TGGGCCTCGTTCCCCTGGAG
COL4A6	Human	TGATTTGGATGATTGTGTGACT/GACTGATTAGGCGATTAGGAAGA
COL5A1	Human	GCCCGGATGTCGCTTACAG/AAATGCAGACGCAGGGTACAG
PLOD3	Human	GCGCCAGTGGAAGTACAAGGAT/CACTTCATCTAAAGCCCCGTTGA
LASP1	Human	GTATCCCACGGAGAAGGTGA/TGTCTGCCACTACGCTGAAA
NIT1	Human	GTGTGCCAGGTAACATCGAC/AGGGTCCCGTGCAATGAAG
TPI1	Human	TGTGTGGGCCATTGGTACTG/CCGCATCAGAGACGTTGGA
G6PD	Human	GGCAACAGATACAAGAACATGAA/CCCTCATACTGGAAACCCACT
FBXL15	Human	CTGGTCTCCGCAGCCTCTCTC/AGGTCAAGGTGGTGGAGTTCTGG
LGALS8	Human	TTGAGATCGTGATTATGGTGCT/ATCCTGTGGCCATAGAGCAG
LYZ	Human	TGCTGCAAGATAACATCGCTG/CCATGCTCTAATGCCTTGTGG
IGHV3-49	Human	GGGGAGGCTTGGTACAGC/AGGCGATGCTTTTGGAATCA
GALNT16	Human	CCACCAGCGTCATCATCACCTTC/ATCAAGTTGGCAGGAGTTCGGTTC
SEMA3A	Human	ATCAGTGGGTGCCTTACCAA/GCCAAATGTTTTACTGGGACA
SERBP1	Human	GAGGACGAGGTGGACGTG/GGAGCAGAAGCACTTGACTTG
DCAF7	Human	CACGGCAAACGGAAGGAGAT/GACGCCTTTTGTGTCAGGGA
XRCC6	Human	TCATGGCAACTCCAGAGCAG/AACCTTGGGCAATGTCAGGT
P4HB	Human	GGAATGGAGACACGGCTTC/TTCAGCCAGTTCACGATGTC
MMP-9	Mouse	CTTCACCGGCTAAACCACCT/TGTCCCTAACGCCCAGTAGA
β-actin	Mouse	AGTGTGACGTTGACATCCGT/GCAGCTCAGTAACAGTCCGC
MMP-9 WT-F1/R1	Mouse	GTGGGACCATCATAACATCACA /CTCGCGGCAAGTCTTCAGAGTA
MMP-9 Mutant-F2/R2	Mouse	CTGAATGAACTGCAGGACGA /ATACTTTCTCGGCAGGAGCA

Methods and materials

Cell lines

HEK293T (CRL-11268, ATCC), HEp-2 (CCL-23, ATCC), and A549 (CCL-185, ATCC) cells were maintained in Dulbecco's Modified Eagle Medium (DMEM; SH30022.01, HyClone) supplemented with 10% fetal bovine serum (FBS; 10099141, Gibco), 100 IU/mL penicillin and 100 μg/mL streptomycin (15140122, Gibco), and 20 mM HEPES (15630080, Gibco). Primary human bronchial epithelial cells (HBEC; CP-H009, Procell) were cultured in HBEC-specific completed medium (CM-H009, Procell). All cell lines were incubated at 37°C in a humidified 5% CO_2 atmosphere and were routinely confirmed to be mycoplasma-free through monthly PCR testing. HEK293F suspension cells were propagated in serum-free SMM 293-TII Expression Medium (M293TII, Sino Biological Inc.) using orbital shakers (160 rpm) under equivalent temperature and CO_2 conditions.

Mice

Female BALB/c mice at the age of 8 weeks were purchased from GemPharmatech Co., Ltd (Nanjing, China). Female FVB/N MMP-9-deficient mice (MMP-$9^{-/-}$; JAX stock #004104) and wild-type FVB/N controls, originally obtained from the Jackson Laboratory, were provided by the Model Animal Research Center of Nanjing University (Nanjing, China)(Coussens et al., 2000). Genotype identification was performed with PCR (**Figure S7**). The mice were housed in a specific pathogen-free environment under standard conditions. All animals were group-housed (3-5 animals

per cage) under a standard 12-hour light/12-hour dark cycle, and all experiments were performed during the light cycle. All animal experiments were approved by the Ethics Committee of Guangzhou National Laboratory Animals.

Viruses

RSV A2 (HEp-2) and RSV B18537 (HEp-2) were propagated and stored at -80°C, respectively. Virus titers were determined with 10-fold serial dilutions in confluent HEp-2 cells in 96-well microtiter plates. Three to four days after inoculation, a cytopathic effect (CPE) was scored, and the Reed-Muench formula was used to calculate the $TCID_{50}$. All the infection experiments were performed at BSL-2 in the Guangzhou National Laboratory.

Compounds, reagents and antibodies

Doxycycline, Doxycycline hyclate, JNJ0966, Gartanin, PC786, ZED-1227, Cystamine, Bacitracin, E64FC26, and ML359 were obtained from MedChemExpress (MCE).

Protein G agarose, fast flow (16-266) used for immunoprecipitation was obtained from Millipore. DAPI (62248) and Pierce Pull-Down PolyHis Protein: Protein Interaction Kit (21277) were sourced from Thermo Fisher Scientific.

The antibodies used in this study are listed: Respiratory Syncytial Virus antibody (FITC) (GTX36375, GeneTex), Mouse anti-RSV F antibody (ab43812, Abcam), Mouse anti-RSV M2-1 antibody (ab94805, Abcam), Mouse anti-RSV N antibody

(ab94806, Abcam), Rabbit anti-MMP-9 antibody (ab283575, Abcam), Rabbit anti-MMP-9 antibody (ab76003, Abcam), Rabbit anti-MMP-9 antibody (#13667, CST), Rabbit anti-HA tag antibody (#3724, CST), Rabbit anti-FLAG tag antibody (#14793, CST), Mouse anti-HA tag antibody (H9658, Sigma-Aldrich), Mouse anti-β-actin antibody (66009-1-Ig, Proteintech), Mouse anti-GAPDH antibody (60004-1-Ig, Proteintech), Rabbit anti-IgG antibody (30000-0-AP, Proteintech), Mouse anti-IgG antibody (B900620, Proteintech), Mouse anti-TGase2 antibody (sc-48370, Santa Cruz); Rabbit anti-Phospho-NF-κB p65 (Ser536) (93H1) antibody (#3033, CST); Rabbit anti-NF-κB p65 antibody (sc-109, Santa Cruz); Peroxidase AffiniPure Goat Anti-Rabbit IgG (H+L) (111-035-003, Jackson ImmunoResearch), Peroxidase AffiniPure Goat Anti-Mouse IgG (H+L) (115-035-146, Jackson ImmunoResearch), Alexa fluor 488-labeled donkey anti-mouse IgG (A-21202, Thermo Fisher Scientific), Alexa fluor 568-labeled donkey anti-rabbit IgG (A-10042, Thermo Fisher Scientific), Cy3-conjugated anti-mouse IgG (B100801, BaiQianDu Biotechnology).

Pseudotyped RSV production and infection

RSV F/G/SH-pseudotyped lentiviruses were generated in HEK293T cells co-transfected with RSV F (1.2 μg), G (0.5 μg), SH (0.5 μg), or pcDNA3.1 empty vector, psPAX2 (0.4 μg), and pWPXLd-Firefly-Luc reporter (0.4 μg) using polyethylenimine (PEI, 24765, Polysciences)(Haid et al., 2015). Pseudovirion-containing supernatants were centrifuged (5,000 × g, 10 min) to harvest pseudovirions. HEp-2 cells treated with 5 μM doxycycline hyclate/JNJ0966 or DMSO were infected with pseudoviruses.

Luciferase activity was quantified by One-Lite Luciferase Assay System (DD1203-03, Vazyme) at 48 hpi and normalized to DMSO controls. Biological triplicates with technical duplicates confirmed data robustness.

Plasmid construction

The codon-optimized gene encoding DS-Cav1 and MMP-9 was synthesized by GenScript. RSV protein-expressing plasmids were codon-optimized for enhanced expression efficiency and synthesized by Beijing Tsingke Biotech Co., Ltd. MMP-9 gene mutants with amino acid substitutions were generated using KOD-Plus-Neo (KOD-401, TOYOBO). The cDNA for TGM2 was amplified from HEp-2 cells infected with RSV A2, with an HA tag at the 5′ end and a FLAG tag at the 3′ end. Specific TGM2 mutants with amino acid substitutions were generated using KOD-Plus-Neo. TGM2 domain deletion mutants, including residues 1-375 (HA-TGM2-44) and residues 376-687 (TGM2-41-FLAG), were obtained by PCR from the TGM2 expression plasmids. To generate vectors expressing proteins with different tags, the open reading frame was excised using restriction enzymes. All plasmids were cloned into the pcDNA3.1 expression vector. The primers used are listed in **Table S1**.

Protein expression and purification

Recombinant plasmids were transfected into HEK293F cells using polyethylenimine (PEI, 24765, Polysciences) at a 1:3 plasmid DNA-to-PEI ratio. Five days post-transfection, supernatants were harvested, clarified by centrifugation and filtration, and subjected to initial purification via Ni Sepharose^{TM} 6 Fast Flow (17531801,

Cytiva) with elution in 1 × PBS (pH 7.4) containing 500 mM imidazole. DS-Cav1 and MMP-9 were further purified by size-exclusion chromatography using a Superose 6 10/300 GL column (17517201, Cytiva) equilibrated in 1 × PBS (pH 7.4), followed by concentration to ~10 mg/mL.

RNA-mediated interference

For siRNA knockdown, host gene-targeting siRNAs (sequences provided in **Table S2**) were synthesized by GenePharma. Cells were transfected with 20 nM of siRNA using Lipofectamine RNAiMAX reagent (13778150, Thermo Fisher Scientific) according to the manufacturer's protocol. A non-targeting scrambled siRNA (siNC) served as the negative control.

Cell viability assay

Cell viability was evaluated using a CellTiter-Glo® Luminescent Cell Viability Assay kit (G7571, Promega) according to the manufacturer's instructions. In brief, 2×10^4 cells in 100 μL culture medium were seeded into 96-well plates for 24 h followed by compounds addition. 100 μL of CellTiter-Glo reagent was added to each well after 72 h inoculation. After a 5-minute shaking and 10-minute incubation, luminescence was measured by PerkinElmer Ensight reader (PerkinElmer). The half-cytotoxic concentration (CC_{50}) was assessed in the absence of viruses. The CC_{50} was calculated using nonlinear regression and GraphPad Prism 8.0 software.

High-throughput antiviral screening

The L1100 protease inhibitor library (246 compounds; L1100, TargetMol) was screened against RSV A2 in HEp-2 cells. Compounds (10 mM DMSO stocks) were robotically dispensed 1 h pre-infection at final concentrations of 1, 5, 10 μM. Infection rates (72 hpi) were normalized to PC786 (1 μM; positive control; HY-111813, MCE) and 0.1% DMSO vehicle. Primary hits (>50% CPE reduction + not overtly cytotoxic) underwent dose-response validation (0.00004–10 μM) to generate EC_{50} curves (GraphPad Prism 8.0).

Evaluation of *in vitro* antiviral activity

Compound inhibitor treatments: HEp-2 cells (1.3×10^4/well) or HBEC (1.8×10^4/well) were pretreated with test compounds for 1 h before RSV A2/B18537 infection (MOI=0.1 or 0.3). Viral inhibition was quantified at 72 hpi via FFA. Fixed cells (4% PFA) were permeabilized (0.3% Triton X-100), stained with anti-RSV-FITC (GTX41322, GeneTex; 1:100), and imaged at 4× magnification (EVOS M5000). Fluorescent foci were normalized to nuclear counts (ImageJ software) for EC_{50} determination via four-parameter logistic regression (GraphPad Prism 8.0). Data represent triplicate experiments.

siRNA-mediated knockdown: Cells were transfected with siNC (control), siMMP-9, or siTGM2 for 24 h before RSV infection (MOI=0.2 or 1). Viral titers and syncytia formation were assessed at 72 hpi via focus-forming assay (FFA) and microscopy. Combinatorial siRNA transfections (siMMP-9/siTGM2) and rescue experiments with

MMP-9/TGM2 overexpression plasmids were analyzed similarly.

Exogenous gene expression: HEp-2 cells were transfected for 24 h with plasmids encoding pcDNA3.1 (control), wild-type MMP-9, TGM2, their mutants (MMP-9-E402Q, TGM2-P375A, TGM2 truncations TGM2-44/TGM2-41), or various combinations prior to RSV infection (MOI=0.2 or 1). Viral titers and syncytia formation were assessed at 72 hpi via FFA and microscopy. All experiments included triplicate biological replicates and statistical validation.

Time-of-drug addition analysis

HEp-2 cells were seeded at a density of 1.3×10^4 cells per well in 96-well plates and subjected to three distinct treatment regimens to evaluate the stage-specific antiviral effects of doxycycline (5 μM), doxycycline hyclate (5 μM), JNJ0966 (5 μM), and gartanin (5 μM). All experiments were conducted under standardized infection conditions with RSV at an MOI of 1. Three regimens were applied: Full-cycle: Cells were pre-treated 2 h pre-infection, co-incubated with virus-drug mixture for 2 h, washed with PBS, and maintained in drug-containing medium until 72 hpi. Entry phase: Drugs were present only during the 2 h viral attachment at 37℃; the post-attachment medium was drug-free. Post-entry: Drugs were introduced after 2 h of infection (virus removed) and maintained until the endpoint. The cytopathic effect reduction was quantified by ImageJ software. DMSO controls followed full-cycle treatment protocols.

Viral genomic replication assay

HEp-2 cells were infected with RSV (MOI=1, 37°C) and treated with compounds (5 μM doxycycline hyclate/JNJ0966) or DMSO control at three phases: pre- (-2–0 h), during (0–2 h), and post-infection (2–16 h). Total RNA was extracted at 22 hpi for RT-qPCR analysis using primers targeting the RSV *F* gene and *β-actin* **(Table S3)**. Viral RNA levels were normalized to *β-actin* via the $2^{(-\Delta\Delta Ct)}$ method. Inhibition rates (%) were calculated as [1 - (treated/control RNA level)] × 100. Experiments included triplicate replicates.

Viral assembly and egress assay

RSV-infected HEp-2 cells (MOI=1, 37°C) were triple PBS-washed and maintained in a drug-free medium. Test compounds (5 μM doxycycline hyclate/JNJ0966) or DMSO control were administered at 10/16 hpi. Supernatants collected at 22 hpi were analyzed for viral titers via FFA. Experiments included three biological triplicates.

Viral multistep growth kinetics

Confluent HEp-2, A549, or HBEC cells pretreated with 5 μM doxycycline hyclate, JNJ0966, DMSO (vehicle control), or left untreated (no drug) for 2 h at 37°C were infected with RSV (MOI=1) under continuous drug exposure for 2 h. Post-adsorption, thrice PBS-washed cells were maintained in drug-supplemented, vehicle-containing, or drug-free medium at 37°C. Intracellular RNA extracted at specified hpi using TRIzol was analyzed for RSV *F* and *β-actin* mRNA via RT-qPCR (primers in **Table**

S3). Viral titers in supernatants were quantified by $TCID_{50}$ assay. Viral replication kinetics were normalized to untreated and DMSO controls, with biological triplicates and technical duplicates ensuring statistical robustness.

Virus-free syncytia assay

HEK293T cells were seeded in 12-well plates at a density of 2×10^5 cells per well and cultured for 20 h. Subsequently, the cells were transfected with RSV F plasmid along with varying concentrations of MMP-9, MMP-9-E402Q plasmid, siNC, or siMMP-9. And cell-cell fusion was observed 48 h using microscopy.

Co-immunoprecipitation

HEK293T cells transfected with epitope-tagged plasmids were harvested 48 hpi. HEp-2 cells co-transfected with plasmids and siRNAs for 24 h were infected with RSV A2 (MOI=1) for 72 h. Cells were washed with ice-cold PBS and lysed in Western/IP lysis buffer (P0013, Beyotime) containing 1 mM PMSF for 30 min at 4°C. Lysates were centrifuged (10,000 × g, 10 min, 4°C), with 10% supernatant retained as input. The remaining lysates were incubated with specific antibodies (1 h, 4°C), followed by protein G agarose beads (16-266, Millipore, 16 h, 4°C). Beads were washed five times with ice-cold lysis buffer (500 × g, 1 min, 4°C) and bound proteins eluted in 2× SDS loading buffer (P0288, Beyotime) for immunoblotting.

Immunoblotting

Cells were harvested, washed with PBS, and lysed in ice-cold Western/IP lysis buffer

(P0013, Beyotime) supplemented with 1 mM PMSF for 30 min. Lysates were centrifuged (10,000 × g, 10 min, 4°C), and supernatants were quantified using an Enhanced BCA Assay Kit (P0010, Beyotime). Proteins (30 μg/lane) were mixed with 5 × SDS-PAGE loading buffer (P0015L, Beyotime), boiled (100°C, 10 min), and separated on 4–20% or 10% Bis-Tris precast gels (SLE020/SLE022, Smart-Lifesciences). Electrophoresed proteins were transferred to PVDF membranes (ISEQ00010, Millipore) via wet transfer. Membranes were blocked with 5% non-fat milk in TBST (0.1% Tween-20) for 1 h, then incubated with primary antibodies (1 h at RT or overnight at 4°C). After three 10-min TBST washes, membranes were incubated with HRP-conjugated secondary antibodies (Jackson ImmunoResearch, 1 h, RT). Signals were developed using enhanced chemiluminescence following final TBST washes. Band intensities were quantified with a FluorChem HD2 system (Alpha Innotech) and normalized to β-actin or GAPDH controls.

His-Tag pull-down assay

Protein-protein interactions were investigated using the Pierce Pull-Down PolyHis Protein: Protein Interaction Kit (21277, Thermo Fisher Scientific) with modifications. RSV A2-infected cell lysates (1 mL) were prepared in Pierce lysis buffer supplemented with protease inhibitor cocktail (P1006, Beyotime). Cleared supernatants were used in the next step. Recombinant His-tagged MMP-9 (150 μg) was immobilized on pre-equilibrated HisPur™ Cobalt Resin for 2 h at 4°C. After five washes with wash buffer, add up to the prepared cleared supernatants. After five

washes with wash buffer, bound complexes were eluted with an elution buffer containing 290 mM imidazole and subjected to LC-MS/MS analysis (Orbitrap Exploris 480, Thermo Fisher Scientific) at the Guangzhou National Laboratory Protein Analysis Facility. Raw files were processed using Proteome Discoverer 2.5 (Thermo Fisher Scientific) against the UniProt database.

RNA-seq analysis

HEp-2 cells were infected with RSV A2 at an MOI of 1, with uninfected cells serving as the mock control. At 72 hpi, total RNA was extracted. Strand-specific cDNA libraries were prepared with the NEBNext® Ultra™ II RNA Library Prep Kit for Illumina® (E7770S, NEB) and sequenced on an Illumina platform (Beijing Tsingke Biotech Co., Ltd.). Differential expression analysis of two groups was performed using the DESeq2 R package (1.26.0). DESeq2 provides statistical routines for determining differential expression in digital gene expression data using a model based on the negative binomial distribution. The resulting P values were adjusted using the Benjamini and Hochberg's approach for controlling the false discovery rate. Genes with $FDR < 0.05$ & $|log2(foldchange)| \geq 1$ found by DESeq2 were assigned as differentially expressed. Functional annotation of DEGs was performed through the Kyoto Encyclopedia of Genes and Genomes (KEGG) pathway analysis.

***In vitro* MMP-9 activity assay**

MMP-9 proteolytic activity was quantified fluorometrically using the substrate MCA-Pro-Leu-Gly-Leu-DPA-Ala-Arg-NH_2 (HY-131498, MCE). Recombinant MMP-9

(HY-P73300, MCE) was activated by incubating 100 μg/mL protein with 1 mM p-aminophenylmercuric acetate (APMA; HY-148905, MCE) in TCNB buffer (50 mM Tris, 10 mM $CaCl_2$, 150 mM NaCl, 0.05% Brij-35, pH 7.5) at 37°C for 24 h. Activated enzyme was diluted to 0.4 ng/μL (final reaction concentration: 0.2 ng/μL) in assay buffer. Substrate stock (2 mM in DMSO) was diluted to 20 μM in TCNB buffer. Reactions were initiated by combining 50 μL activated MMP-9 with 50 μL substrate (final volume: 100 μL) in black 96-well plates. Control wells contained substrate without enzyme to correct for autohydrolysis. Fluorescence kinetics (excitation/emission: 320/405 nm) were monitored every 20 s for 4 min at 37°C using a PerkinElmer Ensight reader (PerkinElmer).

In vitro **MMP-9 cleavage assay**

TGM2 (2 μg, MCE) was incubated with 200 ng of activated MMP-9 (MCE) in 50 mM Tris buffer (pH 7.5) containing 1 mM $CaCl_2$, 50 mM NaCl, and 1 mM $ZnCl_2$ at 37℃ for 18 h. Samples were further separated by SDS-PAGE and detected by Coomassie Brilliant Blue, or were purified by size-exclusion chromatography with a Superdex™ Increase 200 10/300 GL column (28990944, Cytiva) to obtain the target protein (TGM2-44).

Reduction and denaturation of proteins

Reduced DS-Cav1 was prepared by the method described previously(Hasegawa et al., 2003). Briefly, purified DS-Cav1 (20 mg/mL) proteins were dissolved in 0.1 M Tris/HCl buffer (pH 8.6) containing 0.15 M DTT and 6 M guanidine/HCl. They were

incubated at room temperature for 18 h. Proteins were then separated from small molecules by Sephadex G-25 column (SEC018C51, Smart-lifesciences) pre-quilibrated with 200 mM Tris/HCl buffer (pH 7.5). The eluted rdDS-Cav1 was aliquoted and stored at -80°C until use.

Determination of PDI activity

PDI activity was assessed by the measurement of free SH groups of RSV F protein (DS-Cav1). Reaction mixtures containing 200 mM Tris-HCl (pH 7.5), TGM2 or its proteolytic fragment TGM2 (TGM-44), and rdDS-Cav1 were incubated at 37°C under anaerobic conditions achieved by nitrogen purging. Aliquots collected at designated time points were immediately mixed with 0.25 mM DTNB (S0138M, Beyotime) to terminate thiol-disulfide exchange. Absorbance at 412 nm was measured using a PerkinElmer Ensight reader (PerkinElmer).

N-terminal sequencing of TGM2 fragment

TGM2 (10 μg) was incubated with activated MMP-9 (0.5 μg) in 50 mM Tris-HCl buffer (pH 7.5) containing 1 mM $CaCl_2$, 50 mM NaCl, and 1 mM $ZnCl_2$ at 37 °C for 18 h. The reaction was quenched by boiling in the SDS-sample buffer. Proteolytic products were resolved on 10% gels and electrophoretically transferred to a polyvinylidene difluoride membrane via wet transfer. Protein bands of interest were stained, excised, and subjected to Edman degradation-based NH_2-terminal microsequencing at Beijing Biotech Pack Scientific Co., Ltd.

Bio-layer interferometry

Binding kinetics between MMP-9 and its interactors (RSV F protein [DS-Cav1] and TGM2) were quantified using a Sartorius Octet R4 system. Anti-penta-HIS (HIS1K) biosensors (Sartorius) were hydrated in phosphate-buffered saline (pH 7.4; VWR) for 10 min. Proteins (DS-Cav1: 20 μg/mL; TGM2: 10 μg/mL; MCE) were immobilized via 300-s loading in 1 × kinetics buffer (Sartorius), preceded by a 60-s baseline equilibration. MMP-9 (MCE) binding was assessed across two concentration ranges: low (0–5 μM, 7-point serial dilution) and high (0–100 μM, 7-point series), with 300-s association/dissociation phases separated by secondary baseline stabilization. Reference sensors without immobilized ligands corrected for nonspecific interactions.

For TGM2/RSV F interaction studies, biotinylated DS-Cav1 (prepared using a biotinylation kit; G-MM-IGT, Genemore) was coupled to streptavidin (SA) biosensors (Sartorius) at 20 μg/mL. TGM2 binding was evaluated across 0–100 μM (7 concentrations). All assays included triplicate technical replicates.

Data were processed using Octet Analysis Studio v13.0 with a 1:1 global binding model. Kinetic parameters (K_D) were derived after reference subtraction and drift correction. System suitability criteria required χ^2 values <10% and residuals within ±0.1 nm.

Immunofluorescent assay

Immunofluorescence analysis was conducted as follows: HEp-2 cells grown on glass-

bottom dishes were transfected with specified plasmids using TransIT-X2 (MIR 6000, Mirus Bio). At 24 h post-transfection, cells were infected with RSV A2 (MOI=1) for 72 h. HEK-293T cells underwent parallel transfection with Lipofectamine 2000 (11668019, Thermo Fisher Scientific). Both cell types were fixed with 4% paraformaldehyde (30 min, RT) at 48 h post-transfection (HEK-293T) or at 72 h post-infection (HEp-2). Cells were permeabilized with 0.3% Triton X-100 (15 min), blocked with 5% BSA (B824162, Macklin; 1 h), and incubated overnight at 4°C with primary antibodies: anti-RSV F, anti-MMP-9, or anti-FLAG/HA. After PBS washes, samples were stained with Alexa Fluor 488-conjugated donkey anti-mouse IgG or Alexa Fluor 568-conjugated donkey anti-rabbit IgG (1 h, RT), followed by DAPI nuclear counterstain (62248, Thermo Fisher Scientific). Imaging was performed using a Nikon A1 confocal microscope (NIKON CORPORATION) with a 60× oil-immersion objective.

Enzyme-linked immunosorbent assay

Human and murine MMP-9 concentrations were quantified using an established ELISA assay (human MMP-9; DMP900, R&D Systems and mouse MMP-9; EMC013, NeoBioscience) following the manufacturer’s protocol. The samples stored at −80°C were thawed to room temperature, and wherever required, dilutions were made (1:20 for MMP-9). Assay buffer (100 μL) and samples (100 μL for MMP-9) were added to the wells of a microtiter plate coated with respective specific MMP-9 antibodies, followed by incubation (2 h, RT) on a horizontal orbital microplate shaker. After

incubation, plates were washed with buffer three times for 5 min each. Then 200 μL of respective conjugates were added to respective plates and then incubated for 2 h at room temperature. Further, 200 μL of substrate solution was added to each well and incubated for 30 min at room temperature in the dark. Finally, after the addition of 50 μL of stop solution, absorbance at 540 nm was subtracted from the reading at 450 nm by PerkinElmer Ensight reader, and analyte concentrations were calculated from the respective standard curves.

Animal experiments

Thirty 8-week-old female BALB/c mice were obtained from GemPharmatech Co., Ltd (Nanjing, China) and maintained under specific pathogen-free conditions. Doxycycline hyclate and JNJ0966 were administered via oral gavage 2 hours prior to viral challenge. Treatment groups received either 60/180 mg/kg doxycycline hyclate or 20/60 mg/kg JNJ0966, dissolved in 200 μL vehicle solution (5% DMSO/20% hydroxypropyl-beta-cyclodextrin) twice daily (BID) for 4 days. Control animals received the vehicle solution alone. On infection day, mice were anesthetized with isoflurane and intranasally inoculated with 3×10^6 plaque-forming units (PFU) of RSV A2. All animals were euthanized at day 4 post-infection (dpi) for tissue collection. Sample detection was carried out according to the method described in our previous study(Yang et al., 2024). The right lung tissues were homogenized in 1,000 μL PBS using a tissue grinder. Viral titers were quantified via FFA using 100 μL of homogenate. The left lungs were fixed in 4% formaldehyde, paraffin-embedded,

sectioned, and subjected to hematoxylin and eosin (H&E) staining or immunolabeling. Two blinded pathologists independently scored histopathological changes on a 0-4 severity scale.

Age-matched 8-week-old female MMP-9 knockout (*MMP-9*$^{-/-}$, n=8) and wild-type (n=8) mice were anesthetized and intranasally challenged with 3×10^{6} PFU RSV A2. Body weights were monitored daily. Lungs were harvested at 4 dpi for virological and transcriptomic analyses. Viral loads were determined as focus-forming units per gram (FFU/g) using HEp-2 cells. Histological processing followed standard protocols for H&E staining, immunohistochemical staining, and immunolabeling. RNA sequencing libraries were prepared from lung tissue extracts for transcriptomic profiling.

For the live virus model, thirty-two female BALB/c mice at the age of 8 weeks were purchased from GemPharmatech Co., Ltd (Nanjing, China). Mice were inoculated intranasally with 5× 10^{5} PFU /mouse of RSV A2 or an equivalent volume of PBS. At different time points post-inoculation, the lung tissues were collected for virological analysis and assessment of host MMP-9 expression.

Histology and immunolabelling

Histopathological evaluation, immunohistochemical staining, and indirect immunofluorescence (IF) staining were performed following standardized protocols. Lung tissues were fixed in 4% paraformaldehyde (24 h), paraffin-embedded, and sectioned (3.5 μm). H&E staining used Gill's hematoxylin/eosin (B1002, BaiQianDu Biotechnology). For immunofluorescence, deparaffinized sections underwent antigen

repair (EDTA antigen repair buffer, pH 9.0), blocking (10% goat serum), then sequential incubation with mouse anti-RSV-N serum (1:100, 4°C/overnight), Cy3-conjugated anti-mouse IgG (1:200, B100801, BaiQianDu Biotechnology; 1 h/RT), and DAPI (1:1,000, 10 min). Imaging utilized a 3D HISTECH Pannoramic MIDI scanner and an Olympus FV1200 confocal microscope with standardized parameters. For the IHC staining, the rabbit anti-MMP-9 antibody (1:100) and HRP-conjugated goat anti-Rabbit IgG antibody were applied as primary and secondary antibodies, respectively. Subsequently, samples were washed, and the enzyme substrate was added to the sections for 20 min at RT. Images were acquired using an Eclipse E100 microscope (Nikon) and an LG-S80 (Servicebio).

Quantification and statistical analysis

All data were analyzed using GraphPad Prism 8.0 software and represented as mean ± SD in the figures. Statistical comparison between different groups was performed using the corresponding statistical analysis labeled in the figure legends, combining several experiments. *p*-values were calculated, and statistical significance was expressed as highly significant with $*p < 0.05$, $**p < 0.01$, $***p < 0.001$, ns, not significant.

References

Coussens, L.M., Tinkle, C.L., Hanahan, D., Werb, Z., 2000. MMP-9 supplied by bone marrow-derived cells contributes to skin carcinogenesis. Cell 103, 481–490. https://doi.org/10.1016/s0092-8674(00)00139-2

Haid, S., Grethe, C., Bankwitz, D., Grunwald, T., Pietschmann, T., 2015. Identification of a Human Respiratory Syncytial Virus cell entry inhibitor by using a novel lentiviral pseudotype system. J. Virol. 90, 3065–3073. https://doi.org/10.1128/JVI.03074-15

Hasegawa, G., Suwa, M., Ichikawa, Y., Ohtsuka, T., Kumagai, S., Kikuchi, M., Sato, Y., Saito, Y., 2003. A novel function of tissue-type transglutaminase: protein disulphide isomerase. Biochem. J. 373, 793. https://doi.org/10.1042/BJ20021084

Yang, Q., Xue, B., Liu, F., Lu, Y., Tang, J., Yan, M., Wu, Qiong, Chen, R., Zhou, A., Liu, L., Liu, J., Qu, C., Wu, Qingxin, Fu, M., Zhong, J., Dong, J., Chen, S., Wang, F., Zhou, Y., Zheng, J., Peng, W., Shang, J., Chen, X., 2024. Farnesyltransferase inhibitor lonafarnib suppresses respiratory syncytial virus infection by blocking conformational change of fusion glycoprotein. Signal Transduct. Target. Ther. 9, 144. https://doi.org/10.1038/s41392-024-01858-5
